# Real-time sewage surveillance for SARS-CoV-2 in Dhaka, Bangladesh versus clinical COVID-19 surveillance: a longitudinal environmental surveillance study (December, 2019–December, 2021)

**DOI:** 10.1016/S2666-5247(23)00010-1

**Published:** 2023-06

**Authors:** Elizabeth T Rogawski McQuade, Isobel M Blake, Stephanie A Brennhofer, Md Ohedul Islam, Syed Shahnewaj Siraj Sony, Tonima Rahman, Md Hamim Bhuiyan, Sabrina Karim Resha, Erin G Wettstone, Lauren Hughlett, Claire Reagan, Sarah E Elwood, Yoann Mira, Ayesha S Mahmud, Kawsar Hosan, Md Raihanul Hoque, Md Masud Alam, Mahbubur Rahman, Tahmina Shirin, Rashidul Haque, Mami Taniuchi

**Affiliations:** a Division of Infectious Diseases & International Health, University of Virginia, Charlottesville, VA, USA; b Department of Biomedical Engineering, University of Virginia, Charlottesville, VA, USA; c Department of Civil and Environmental Engineering Systems and Environment, University of Virginia, Charlottesville, VA, USA; d Department of Epidemiology, Emory University, Atlanta, GA, USA; e MRC Centre for Global Infectious Disease Analysis, School of Public Health, Imperial College London, UK; f icddr,b, Dhaka, Bangladesh; g Novel-T, Geneva, Switzerland; h Department of Demography, University of California at Berkeley, Berkeley, CA, USA; i a2i, Dhaka, Bangladesh; j Department of Economics, Jahangirnagar University, Dhaka, Bangladesh; k Institute of Epidemiology, Disease Control and Research, Dhaka, Bangladesh

## Abstract

**Background:**

Clinical surveillance for COVID-19 has typically been challenging in low-income and middle-income settings. From December, 2019, to December, 2021, we implemented environmental surveillance in a converging informal sewage network in Dhaka, Bangladesh, to investigate SARS-CoV-2 transmission across different income levels of the city compared with clinical surveillance.

**Methods:**

All sewage lines were mapped, and sites were selected with estimated catchment populations of more than 1000 individuals. We analysed 2073 sewage samples, collected weekly from 37 sites, and 648 days of case data from eight wards with varying socioeconomic statuses. We assessed the correlations between the viral load in sewage samples and clinical cases.

**Findings:**

SARS-CoV-2 was consistently detected across all wards (low, middle, and high income) despite large differences in reported clinical cases and periods of no cases. The majority of COVID-19 cases (26 256 [55·1%] of 47 683) were reported from Ward 19, a high-income area with high levels of clinical testing (123 times the number of tests per 100 000 individuals compared with Ward 9 [middle-income] in November, 2020, and 70 times the number of tests per 100 000 individuals compared with Ward 5 [low-income] in November, 2021), despite containing only 19·4% of the study population (142 413 of 734 755 individuals). Conversely, a similar quantity of SARS-CoV-2 was detected in sewage across different income levels (median difference in high-income *vs* low-income areas: 0·23 log_10_ viral copies + 1). The correlation between the mean sewage viral load (log_10_ viral copies + 1) and the log_10_ clinical cases increased with time (*r* = 0·90 in July–December, 2021 and *r*=0·59 in July–December, 2020). Before major waves of infection, viral load quantity in sewage samples increased 1–2 weeks before the clinical cases.

**Interpretation:**

This study demonstrates the utility and importance of environmental surveillance for SARS-CoV-2 in a lower-middle-income country. We show that environmental surveillance provides an early warning of increases in transmission and reveals evidence of persistent circulation in poorer areas where access to clinical testing is limited.

**Funding:**

Bill & Melinda Gates Foundation.

## Introduction

Throughout the COVID-19 pandemic, inequities in access to diagnostic testing for SARS-CoV-2 have limited the accuracy of clinical case surveillance for characterising population-level disease burden and dynamics.^1^ These data are critical for the appropriate deployment of resources and targeting of interventions to areas where disease burden is highest or increasing. Limitations to case data are amplified in low-resource settings where disease burden is often inversely associated with testing access, and therefore passively reported clinical testing data are biased. Furthermore, asymptomatic cases that contribute to transmission are largely missed by clinical case surveillance.^2^ Delays between case onset, testing, and results reporting further limit the utility of clinical surveillance, and these lags can be longer among populations with higher barriers to testing.^3^

Environmental surveillance for SARS-CoV-2 provides an opportunity to supplement traditional clinical surveillance by tracking disease burden at a population level. Because SARS-CoV-2 viral RNA can be detected in human faeces,^4^, ^5^, ^6^, ^7^ wastewater is a composite sample that effectively pools faeces from both symptomatic and asymptomatic individuals living in the catchment areas of environmental surveillance sites.^4^, ^8^ Active surveillance of wastewater for the presence and quantity of SARS-CoV-2 effectively estimates population-level burden without bias in test-seeking behaviour or access to testing.

**Research in context**

**Evidence before this study**

We searched PubMed for manuscripts published from March 1, 2020, to Jan 8, 2023, using the search terms “((((environmental surveillance) OR (wastewater epidemiology)) AND (SARS-CoV-2)) AND (COVID-19)) AND (low income)”. We identified 39 publications, of which five wastewater surveillance studies were from low-income or middle-income countries. Of these, three surveillances were based in large cities with formal sewage systems or used samples from wastewater plants. The remaining studies were cross-sectional studies in which the catchment populations were not well defined.

**Added value of this study**

In this study, we implemented environmental surveillance for SARS-CoV-2 in Dhaka, Bangladesh, where samples were mostly collected from informal sewage systems. Before our study, most environmental surveillance systems for SARS-CoV-2 were used as a public health tool in high-income countries with formal sewage networks or in low-income and middle-income countries at wastewater treatment plants. Here, we demonstrate an environmental surveillance use case in a converging informal sewage network in a lower-middle-income country where most of the sewage is released untreated into the rivers. First, we mapped the sewage network through systematically blue line tracing and digitising every informal and formal sewage line to create an interactive map to estimate catchment population and area. Using the interactive map, 37 environmental sites were selected across three areas of Dhaka and sampled weekly to quantitatively detect the spatiotemporal SARS-CoV-2 viral burden. Working closely with public health stakeholders in Bangladesh, we received weekly geolocated COVID-19 case data. Our findings are shared weekly with the Bangladeshi national COVID-19 task force for mitigation efforts via a weekly report and a dashboard that is publicly available. Secondly, models were developed using the environmental surveillance data and clinical data to understand the association between the quantitative sewage detection of SARS-CoV-2 and the case burden in the study area. Our results suggest that environmental surveillance can detect the rise in cases about 1–2 weeks in advance and provide vital information on community transmission of COVID-19 to public health stakeholders.

**Implications of all the available evidence**

Although most environmental surveillance for SARS-CoV-2 is conducted in areas with formal sewage networks in high-income countries, environmental surveillance can be established in a converging informal sewage network often found in low-income and middle-income countries using blue line tracing, Geographic Information Systems, and reverse transcriptase quantitative PCR for SARS-CoV-2. Complementary surveillance for SARS-CoV-2 to clinical case surveillance is particularly important in low-income and middle-income countries where access to clinical testing is limited or not available, or clinical surveillance is incomplete or altogether missing. Environmental surveillance is a powerful tool that can provide real-time information on COVID-19 transmission on a community level for public health interventions and mitigation efforts.

Environmental surveillance has been used as a complementary surveillance method before COVID-19, for example as part of the Global Polio Eradication Initiative to systematically test sewage for poliovirus.^9^ Environmental surveillance initiatives have now become a standard tool for surveillance of SARS-CoV-2 in high-income countries (HICs) and in some low-income and middle-income countries (LMICs) with formal sewage networks.^7^, ^10^, ^11^, ^12^ In HICs, changes in viral load in sewage have been shown to provide an early warning of changes in transmission in advance of clinical case data, largely due to the excretion of the virus in faeces before the onset of symptoms and the detection of asymptomatic and mild infections that are often under-reported.^5^, ^6^ Because HICs have convergent sewage systems, efficient sampling points are available at sewage treatment plants that represent predictable catchment areas. However, informal sewage networks in LMICs might be affected by changing environmental conditions, and the utility of environmental surveillance in these settings is unknown.

Here, we evaluate the use of environmental surveillance as a supplementary surveillance method for SARS-CoV-2 RNA in Dhaka, Bangladesh, where limited diagnostic testing,^13^ high population density, and weak access to health care has made clinical surveillance for COVID-19 challenging.^14^, ^15^ Firstly, we mapped the informal sewage network in three areas of Dhaka, selecting 37 environmental surveillance sites from which weekly samples were collected. We compared SARS-CoV-2 detection in sewage from 37 catchment areas in eight wards (the smallest administrative unit for urban areas in Bangladesh) with reported clinical cases from December, 2019, to December, 2021, evaluated whether environmental surveillance provides an early warning of changes in transmission in this setting, and explored how this association varied by income level of ward residents.

## Methods

### Site development

Dhaka's sewage network is comprised of primarily informal and some formal converging sewage lines where only 20% of the waste is treated at a wastewater plant while the rest is released directly into the rivers. The Dhaka Water Supply and Sewerage Authority could only provide partial blue lines (mapping of sewage lines) for the formal sewage system in Mirpur Wards 2, 3, 5, and Baridhara/Gulshan areas (appendix pp 8–10, 14). Therefore, it was necessary to blue line trace the rest of the formal sewage lines and all the informal lines and determine the flow direction in each of the lines to create a comprehensive map of the complex wastewater network across the entire study area (22·34 km^2^; appendix pp 3, 8–14). Catchment populations were estimated by integrating information on blue-line sewage lines, digital elevation models, and raster population estimates (WorldPop).^16^ Estimated catchment areas and catchment populations of each environmental site can be viewed online. Details on the catchment estimation are available online.

Surveillance sites were selected across three areas of Dhaka, to represent the diversity of the city in terms of income level and population density. Low-income study areas included were Mirpur Wards 2, 3, and 5; middle-income areas were Mirpur Wards 8, 9, and 10; and high-income areas were Baridhara Ward 18 and Gulshan Ward 19 (appendix pp 15–21). The selected sites had the following site characteristics: (1) estimated catchment population of more than 1000 people, (2) accessible throughout the study, (3) sufficient sewage flow for collection of 6 L samples, (4) pH of sewage around 7, (5) high total dissolved solids of sewage (above 250 mg/L), (6) away from industrial waste inlets, and (7) site access not affected by flooding. Sites in Wards 8, 9, and 10 were selected before the pandemic in 2019 for poliovirus, enteric pathogen, and antimicrobial resistance surveillance. Samples from these sites were retrospectively tested for SARS-CoV-2 from December, 2019, to March, 2020, after which prospective sampling commenced.

### Environmental sample collection

We selected the bag-mediated filtration system (BMFS) to sample sewage since it is easy to collect a large volume of sample in the field and this method provided efficient recovery of a broad spectrum of pathogens such as viruses, bacteria (including *Escherichia coli*), protozoa, and microsporidia in previous studies (data not shown). 6 L grab samples were collected weekly using BMFS from each surveillance site on the same day of the week between 0700 h and 0900 h, when the residents use or release waste into the formal and informal sewage system.^17^ Further details are given in the appendix (p 3).

Using the Aquaread Probe AP-2000-D (Aquaread, Kent, UK), we measured the physiochemical properties of the sample at each collection (appendix p 3). The virus was eluted using beef extract and concentrated via skim milk flocculation, following which nucleic acid extraction was performed (appendix p 3).^18^, ^19^

Reverse transcriptase quantitative PCR (RT-qPCR) for SARS-CoV-2 RNA was conducted to amplify the virus nucleocapsid (N) gene region of the SARS-CoV-2 genome using primer probe mixes for N1 and N2 amplicons that were part of the 2019-nCoV CDC EUA Kit (Integrated DNA Technologies, Coralville, IA, USA; see appendix pp 3–4, including for primer and probe sequences). Positive control for SARS-CoV-2 (2019-nCoV_N_Positive Control RUO plasmid from Integrated DNA Technologies) and negative control (nuclease-free water) were included for each RT-qPCR run.

The samples were tested for faecal indicators HF183 and CrAssphage (human-restricted faecal indicators) using the following published assays: primer-probe HF183/BacR287/BacP234 was used for HF183 detection, and CPQ_056 assay, which included 056F1/056R1/056P1, was used for CrAssphage detection (appendix p 4).^20^, ^21^

### COVID-19 case data

The daily number of samples tested for SARS-CoV-2 and the number of samples that tested positive from March, 2020, to December, 2021 across Dhaka were provided by the Aspire to Innovate (a2i) programme of the Information and Communication Technology Division of the Ministry of Posts, Telecommunications and Information Technology, Bangladesh. We analysed 648 days of case data. a2i aggregates data from the District Health Information Software (DHIS2) platform run by the Management Information System of the Directorate General of Health Services, and provides the case numbers used for clinical surveillance by the Bangladeshi Government. Positive tests were geolocated to the ward level by a2i, using information on the address of residence. We were therefore able to extract the reported clinical cases who resided in our study wards. Negative tests were not routinely geolocated. Geolocated negative tests were only available for 1-week time periods in two months: November, 2020, and November, 2021.

### Statistical analysis

The primary outcome was the transformed cycle threshold (Ct) values from qPCR on the log_10_ scale to estimate the viral load per L of the filtered environmental sample. Each grab of sewage resulted in a different filtered sample volume through BMFS and therefore we calculated the number of viral N1 copies per L of filtered sewage. The analytical performance of real-time RT-qPCR N1 gene assay is given in the appendix (p 4). We present viral load estimates on the following scale: log_10_ ((total sample viral load / volume of filtered sewage sample) + 1) where the + 1 is added to keep the test negatives at 0 on the log scale (the Ct limit of detection was 40 where a Ct value of 40 equated to 0 log_10_ copies). We made these data available in real time on a dashboard alongside clinical cases.

A secondary outcome was to assess the correlation between the mean log_10_ quantity of CrAssphage and HF183 in sewage and (1) log_10_ mean of the estimated catchment area population size (table) and (2) previous day's rainfall and previous 3 days' rainfall (mm; obtained from World Weather Online) to determine whether it was possible to normalise measured concentrations of SARS-CoV-2 to the population contributing to each sewage sample. Fixed-effects linear regression models were used (appendix pp 4–5). CrAssphage and HF183 were not routinely tested for throughout the study. Instead, they were tested for in three different months capturing low (March, 2021), medium (May, 2021), and high (July, 2021) rainfall. We also assessed the correlation between CrAssphage and HF183.

**Table:** Characteristics of the 37 catchment sites throughout eight wards in Dhaka, Bangladesh

	**Site name**	**Dates of surveillance**	**Type of collection**	Geographical size of estimated catchment area (km^2^)*	Population size of estimated catchment area (people)*†	Latitude*	Longitude*
**Low-income**							
Ward 2							
1	Pollobi Women Degree College, Kalshi	November, 2020–December, 2021‡	Open canal	0·18	2911	23·822	90·373
2	Sagufta	November, 2020–December, 2021‡	Wastewater outlet	0·27	3760	23·828	90·377
Ward 3							
1	Paris Road Khal	November, 2020–December, 2021‡	Open canal	0·35	7908	23·812	90·374
3	Avenue-4 Culvert-2	November, 2020–December, 2021‡	Wastewater outlet	0·10	1911	23·817	90·376
Ward 5							
1	Avenue-4 Culvert-1	November, 2020–December, 2021‡	Wastewater outlet	0·17	3803	23·818	90·376
3	22 Floor Garments	November, 2020–December, 2021‡	Open canal	1·36	25 199	23·822	90·374
4	Bauniabad Switch Gate Calvert	November, 2020–December, 2021‡	Wastewater outlet	0·25	3037	23·820	90·383
5	22 Teki Khal	November, 2020–December, 2021‡	Open canal	1·31	30 814	23·813	90·381
6	Mirpur Dohs Link Road-2	November, 2020–December, 2021‡	Open canal	1·55	27 511	23·822	90·383
7	Mirpur Dohs Link Road-1	November, 2020–December, 2021‡	Wastewater outlet	6·63	334 654	23·822	90·383
**Middle-income**							
Ward 8							
1	Switch Gate, Beribadh	December, 2019–December, 2021‡	Wastewater outlet	2·12	137 122	23·800	90·344
2	Momen Dewan Bosti	December, 2019–December, 2021‡	Open canal	0·14	13 777	23·802	90·347
3	Nobaberbagh Bus Stand Manhole	December, 2019–December, 2021‡	Manhole	0·71	76 524	23·806	90·343
4	Muktijoddha Complex Manhole	December, 2019–June, 2020	Manhole	0·31	2450	23·813	90·351
5	Mazar Road Corner Slab	November, 2020–December, 2021‡	Open manhole	0·55	68 850	23·797	90·350
Ward 9							
1	Borobazar Para, Beribadh, Gabtoli	December, 2019–June, 2020	Wastewater outlet	0·14	10 942	23·786	90·339
2	Palpara Ghat, Beribadh Gabtoli	December, 2019–December, 2021‡	Wastewater outlet	0·51	11 578	23·793	90·340
3	Ananda Nagar Block-D	December, 2019–December, 2021‡	Open canal	0·12	4356	23·786	90·344
4	Hanif Bus Counter, Gabtoli	December, 2019–June, 2020	Open manhole	0·08	4443	23·783	90·340
Ward 10							
1	Society Balur Math, Darus Salam	December, 2019–December, 2021‡	Wastewater outlet	0·04	1071	23·778	90·352
2	Boat Stand, Darus Salam Road	December, 2019–December, 2021‡	Wastewater outlet	7·01	585 778	23·777	90·353
3	Btenia Jame Masjid	December, 2019–October, 2020	Open canal	2·13	191 612	23·786	90·350
4	Lalkuthir Bazar, Mirpur Mazar Road	December, 2019–December, 2021‡	Open canal	2·09	181 183	23·788	90·350
5	Shahjadpur Bus Stand, Gabtoli	November, 2020–December, 2021‡	Open canal	4·22	300 420	23·782	90·350
**High-income**							
Ward 18							
1	Road-9 Baridhara Park	December, 2020–December, 2021‡	Wastewater outlet	0·09	1832	23·805	90·418
2	Suvastu Shopping Complex, Badda	December, 2020–December, 2021‡	Open canal	2·05	58 593	23·789	90·425
Ward 19							
1	Brac Center, Gulshan	December, 2020–December, 2021‡	Open canal	0·94	23 550	23·780	90·411
2	Banani-11 Bridge-1	December, 2020–December, 2021‡	Wastewater outlet	0·26	8881	23·790	90·411
3	Banani-11 Bridge-2	December, 2020–December, 2021‡	Wastewater outlet	0·49	18 688	23·790	90·411
4	Karail Bosti	December, 2020–December, 2021‡	Open canal	0·28	4806	23·784	90·407
5	Gulshan Niketon Link Bridge	December, 2020–December, 2021‡	Wastewater outlet	3·23	81 563	23·773	90·415
6	Gulshan-2 Link Bridge-1	December, 2020–December, 2021‡	Wastewater outlet	0·15	2980	23·793	90·410
7	Gulshan-2 Link Bridge-2	December, 2020–December, 2021‡	Wastewater outlet	0·67	21 802	23·794	90·410
8	Gulshan Circle-1 Manhole	February, 2021–December, 2021‡	Manhole	2·88	69 819	23·780	90·417
9	Gulshan Circle-2 Manhole	February, 2021–December, 2021‡	Manhole	1·67	30 478	23·794	90·415
10	Karail Bosti-2	February, 2021–December, 2021‡	Wastewater outlet	0·22	5616	23·787	90·413
11	Karail Bosti-3	February, 2021–December, 2021‡	Wastewater outlet	0·14	3544	23·785	90·414

* Data can be found at https://es.world/country/BGD/Dhaka.

† Population data from Wards 8–10 and Wards 2, 3, 5, 18, and 19 are from 2016 and 2019, respectively.

‡ Sites are still operational as of March, 2023.

We calculated the percent positivity of clinical COVID-19 tests Dhaka-wide from March, 2020, to December, 2021, as a marker of testing access (assuming >5% equates to limited testing).^22^ Another secondary outcome was to test the Pearson correlation between N1 viral load on the log_10_ scale and the weekly log_10_ clinical case data with lags of 0, 1, and 2 weeks both across the whole study population and by ward income level (appendix p 5). This provided an assessment of the overall association between these two time series. Cross-correlations between sewage viral load and clinical case data were estimated via generalised estimating equations^23^ adjusting for ward and temporal autocorrelation (appendix p 5).

All statistical analyses were performed via R software, version 4.0.2.

The study protocol was approved by the Research Review Committee and Ethical Review Committee of the icddr,b and the Institutional Review Boards of the University of Virginia.

### Role of the funding source

The funder of the study had no role in study design, data collection, data analysis, data interpretation, or writing of the report.

## Results

**Figure 1:**
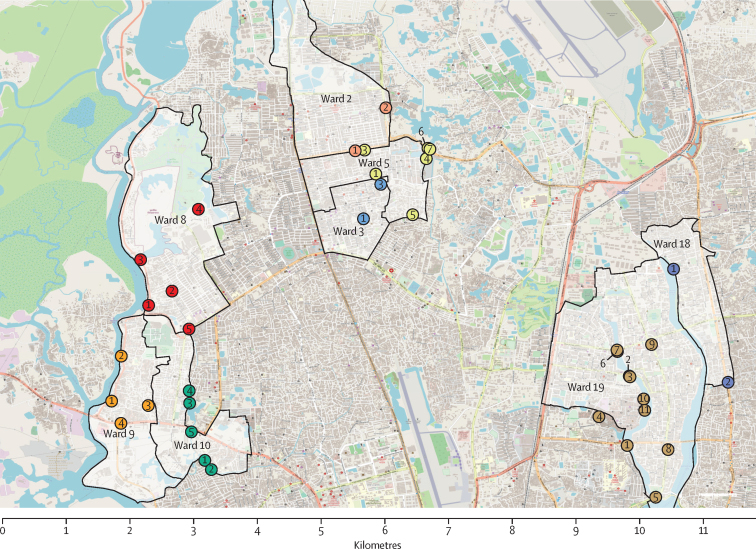
Map of the 37 sewage sites throughout eight wards in Dhaka, Bangladesh Names of catchment sites are outlined in the table.

We tested samples weekly from 37 environmental surveillance sites. The number of sites per ward ranged from two (Ward 3) to 11 (Ward 19), with ten, 14, and 13 sites in the low-income, middle-income, and high-income regions, respectively (figure 1). Surveillance at each site was initiated over an extended period starting in December, 2019 (12 sites), with the most recent sites added in February, 2021 (four sites; table 1). The size of the estimated catchment areas varied by geography and population, ranging from 0·04 to 7·01 km^2^ and 1071 to 585 778 people, respectively. Environmental samples were collected from four types of sources: open canal, wastewater outlet, manhole, and open manhole (ie, a manhole with no lid or covering). Visual depictions of each collection site can be viewed in the appendix (pp 15–21). A total of 2073 sewage samples were analysed.

**Figure 2:**
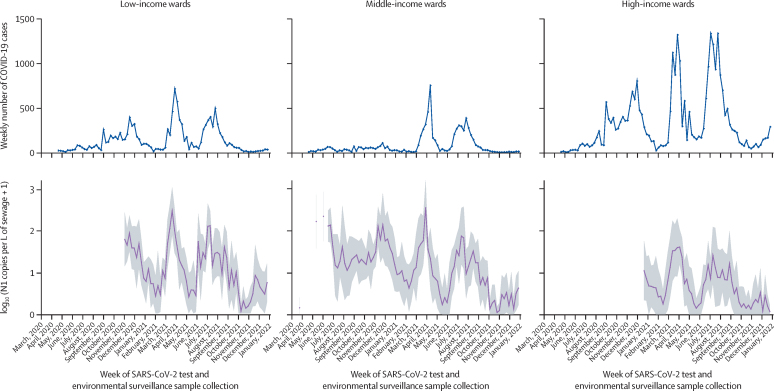
Weekly number of COVID-19 cases and mean log_10_ N1 copies per L of sewage (ribbon 95% CI) by week of SARS-CoV-2 test and environmental surveillance sample collection by study area The clinical case data are from the eight wards where the 37 catchment sites are located. Environmental surveillance data are missing from April, 2020, due to a lockdown throughout Dhaka, and from the second week of June, 2020, due to laboratory restrictions. Environmental surveillance collection schedule was as follows: December, 2019–May, 2020 (monthly), June, 2020–December, 2021 (weekly).

**Figure 3:**
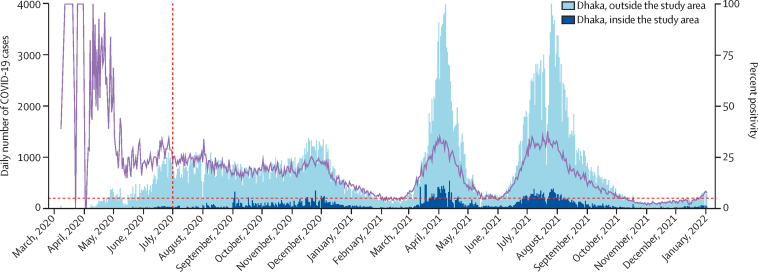
Daily number (blue bars) and percent positivity (purple line) of COVID-19 clinical cases from March, 2020, to December, 2021 in Dhaka, Bangladesh Red horizontal dashed line indicates 5% positivity. Red vertical dashed line indicates when testing became more widely available and percent positivity rates began to stabilise. Darker bars indicate the number of cases reported from the study area.

The first detection of SARS-CoV-2 through environmental surveillance occurred during the week of March 23, 2020 (two of 12 sites), with a mean viral load of 0·47 (log_10_ N1 copies per L of sewage + 1) over the two sites (figure 2). This detection occurred at a time when very few clinical tests were being conducted (figure 3). The viral load of SARS-CoV-2 detected at individual sites ranged from 0 to 3·85 (log_10_ N1 copies per L + 1) throughout the study. However, the mean viral load across sites has not reached 0 since first detection in March, 2020 (figure 2). A similar level of virus was detected across each ward over time (appendix pp 22–23) and overall, by study area (figure 2), whereby three distinct waves of transmission were detected. The absolute difference in mean viral load between high-income and low-income areas over 54 weeks was small (median 0·23 log_10_ N1 copies per L of sewage + 1; IQR 0·46) and on average (mean) was 69% (95% CI 61–77) lower in the high-income wards compared with low-income wards (p<0·0001).

We tested a total of 396 samples for HF183 and CrAssphage. We found no association between the estimated sewage catchment population size and concentrations of 1:100 dilutions of HF183 or CrAssphage, nor between these human faecal contamination markers and rainfall (appendix p 24). Levels of HF183 and CrAssphage across sites were correlated in March (*r*=0·73), May (*r*=0·86), and July (*r*=0·72), 2021.

Between March, 2020, and December, 2021, 488 720 COVID-19 cases were reported in Dhaka, of which 47 683 (9·8%) were from our study areas (figure 3). The first case in Dhaka was reported in March, 2020, whereas the first case in the study area was reported in April, 2020. The percent positivity fluctuated during the first 4 months of the pandemic when testing began. Rapid changes in percent positivity started to stabilise at around 25% in July, 2020, when testing became more available (figure 3). From July, 2020 onwards, the percentage of positive tests Dhaka-wide ranged from 1·7% in November, 2021 (82 of 4955) to 37·7% in July, 2021 (1653 of 4382).

Among the study wards, 26 256 (55·1%) of the 47 683 clinical cases were reported from Ward 19, despite this ward containing only 142 413 (19·4%) of 734 755 people in the study population (appendix p 25). A sample of all tests administered (including negative tests) from the week of Nov 2, 2020, indicated that Ward 19 had 123 times the number of tests per 100 000 individuals compared with Ward 9, which had the lowest number of tests per 100 000 individuals (1603 tests per 100 000 in Ward 19 *vs* 13 tests per 100 000 in Ward 9; appendix p 25). A second sample of testing data from the week of Nov 1, 2021, indicated that Ward 19 had 70 times the number of tests per 100 000 individuals compared with Ward 5, which had the lowest number of tests per 100 000 individuals (429 tests per 100 000 in Ward 19 *vs* six tests per 100 000 in Ward 5).

In each of the three study areas, the quantity of SARS-CoV-2 in sewage increased 1–2 weeks in advance of the major waves of infections (figure 2). SARS-CoV-2 was first detected in sewage in Wards 8 and 9 in March, 2020, whereas clinical cases were not reported until April, 2020 in the study area. Importantly, environmental surveillance identified ongoing community transmission despite very low or zero cases reported in some wards. For example, from April, 2020, to February, 2021, in Ward 10, a low number of clinical cases were reported (mean 15·3 cases per week, SD 11·5, range 1–52) while the viral load detected in sewage was relatively high (mean 1·17 log_10_ N1 copies per L of sewage + 1; SD 0·41, range 0·20–2·05 log_10_ N1 copies per L of sewage + 1). During the week of July 13, 2020, in Ward 9, there were 0 clinical cases despite the environmental sample indicating that there were 1·81 log_10_ N1 copies per L of sewage + 1 throughout the ward.

The strongest correlation between the viral load of the environmental surveillance samples and the number of clinical cases occurred when they were compared from the same week (zero lag; *r*=0·72, p<0·0001; appendix p 26). This correlation held true when further examined by 6-month time intervals. The correlation between environmental viral load and logged case data with no lag was strongest from July to December, 2021 (*r*=0·90, p<0·0001), then January to June, 2021 (*r*=0·80, p<0·0001), and lastly July to December, 2020 (*r*=0·59, p=0·0015; appendix pp 27–29).

Cross correlation of the smoothed (7-day running average) ward-level daily number of logged cases and the weekly environmental surveillance data from July, 2020, to December, 2021, indicated that the highest correlation occurred when the case data lagged the environmental surveillance data by 5 days. A 1 log increase in viral load corresponded to a 0·30 log increase in clinical cases 5 days later (appendix p 30). When this analysis was stratified by 6-month intervals, a 6-day lag in case data was strongest from January to June, 2021, with an increase of 0·34 logged cases per 1 log increase in viral load. However, from July to December, 2020, the lag was longer at 15 days. Results were also stratified by low-income, medium-income, and high-income wards (appendix p 31).

## Discussion

Environmental surveillance for SARS-CoV-2 is feasible and useful for monitoring trends in transmission intensity in a lower-middle-income setting with an informal sewage system. Our findings provide evidence that in a city where clinical testing has been limited, increases in viral load precede changes in cases, such that trends in sewage can provide an early warning for changes in incidence, particularly in advance of major waves of transmission. Environmental surveillance is particularly important in settings with limited or inequitable clinical testing as it provides unbiased sampling at the community level.

Although SARS-CoV-2 has been detected in sewage from populations residing in LMICs, most of these studies collected sewage from formal sewage networks and wastewater treatment plants with a well defined catchment area and population.^24^, ^25^, ^26^, ^27^ Before this study, very few had collected sewage from informal sewage lines where the catchment area and populations are not defined, and these had not assessed temporal trends of sewage viral load with clinical case incidence, stratifying by the income level of the population.^28^ Informal sewage networks are common in cities across many low-income and middle-income countries, and are regularly sampled for poliovirus as part of the Global Polio Eradication Initiative's surveillance strategy, where catchments are hard to define.^9^ What is unique to our study is that we fully traced and digitised all of the informal and formal sewage networks in our study areas to accurately estimate catchment populations, and generated a relatively long time series of environmental surveillance data with which to compare clinical case data from the same population.

Importantly, we found SARS-CoV-2 in similar levels across diverse areas of the city, in contrast to the large variability in clinical cases recorded across wards. This heterogeneity was unrelated to population size and explained by differences in income level and testing intensity. The viral load in sewage suggested the underlying attack rate was more similar across areas than case data suggested. Not surprisingly, the correlation between SARS-CoV-2 and clinical cases was strongest in a high-income ward (Ward 19) with higher rates of clinical testing. We therefore showed that environmental surveillance for SARS-CoV-2 provides a more equitable strategy to conduct surveillance, especially in settings where the availability of clinical testing is limited and differs by geography and population. Our finding that environmental surveillance can provide unbiased information on SARS-CoV-2 transmission when clinical surveillance is heterogeneous is in agreement with results from high-income settings during periods of insufficient clinical testing.^29^

Furthermore, the ability to detect asymptomatic individuals is an important benefit of environmental surveillance since these individuals contribute to transmission and can become more common over time as immunity in the population increases. SARS-CoV-2 was consistently detected in sewage across the three diverse areas of Dhaka, even during periods where few clinical cases were reported. This suggests environmental surveillance can identify ongoing transmission in the absence of high clinical burden, which will be important as the pandemic recedes.

Trends in viral load over time are probably most informative for expected changes in caseloads. We found the case data lagged the sewage data by 5 days, in agreement with other studies comparing such data in HICs.^12^, ^28^, ^30^ It is important to note that cases capture incidence whereas environmental surveillance captures prevalence, and therefore we would expect the decline of peaks in viral load to be slower in sewage than in clinical cases, but for environmental surveillance to capture increases in transmission earlier as it captures asymptomatic infections.

Our study has some limitations. Firstly, informal sewage networks can be heterogeneous across LMICs and the sewage system in Dhaka might not be representative of all LMIC sewage systems. However, environmental surveillance has been standardised for poliovirus across multiple continents and our study demonstrates the wider potential impact environmental surveillance could have in LMICs for monitoring SARS-CoV-2 where clinical case surveillance is limited. Secondly, we did not find a correlation between the estimated catchment population size and human faecal contamination markers. The literature is mixed on the ability to normalise the concentrations of SARS-CoV-2 recovered in wastewater. Some have been able to normalise to CrAssphage where normalisation has improved the association between SARS-CoV-2 recovered in wastewater and clinical cases,^31^ whereas others have been unable to normalise to CrAssphage and other human faecal markers.^30^ These results highlight our inability to predict the absolute number of expected infections from viral load in sewage. Interestingly, however, despite the large amount of rainfall Dhaka experiences in the summer months, we did not find an association between the level of human faecal contamination markers and rainfall. We therefore do not expect the quantity of SARS-CoV-2 to be diluted during these periods and for environmental surveillance to be unaffected by rainfall in Dhaka. Finally, we did not have complete geolocated temporal data on the number of people accessing clinical testing for SARS-CoV-2, and relied on geolocated denominator testing data available for only 1-week periods during November, 2020, and November, 2021. However, it is unlikely that resources for testing in high-income areas would have been diverted to lower-income areas for discrete periods of time.

Work to investigate variants of concern by sequencing the wastewater samples is ongoing. Since sewage surveillance precedes clinical cases, the ability to track emerging SARS-CoV-2 sublineages in sewage would provide more in-depth information on the prevailing and emerging lineages of SARS-CoV-2 before genomic information from clinical cases, as has been demonstrated in the USA.^32^ Understanding the evolution of the virus and identifying strains that are escaping the current vaccines could in turn inform future vaccine designs.

Our data alongside reported clinical case data have been reported weekly to the Government of Bangladesh and in real time on a public dashboard website, allowing for spatiotemporal visualisation of disease prevalence in the eight study wards. The environmental surveillance data presented in the dashboard can be used by public health officials in Bangladesh to monitor disease transmission and aid in making informed and actionable decisions to contain COVID-19 outbreaks. For example, these data could be used to increase localised testing where appropriate, improve messaging to communities, target non-pharmaceutical interventions, and target lockdowns. Furthermore, this surveillance has potential to monitor the impact of vaccination, particularly on the incidence within lower-income populations.

In summary, environmental surveillance is complementary to clinical surveillance, providing an unbiased measure of transmission in advance of clinical testing. This strategy is particularly pertinent to populations in areas where access to clinical testing is inadequate. Because each sewage sample is pooled from many individuals, this type of surveillance is cheaper and requires fewer health professionals than widespread diagnostic testing. Importantly, environmental surveillance promotes health equity by ensuring underserved populations are included in surveillance programmes.

## Data sharing

The sewage qPCR SARS-CoV-2 data can be accessed through written request to the corresponding author with a proposal of secondary data analysis. After approval, data will be sent with a corresponding data dictionary.

## Declaration of interests

We declare no competing interests.
