# Supplementary material for: Real-time sewage surveillance for SARS-CoV-2 in Dhaka, Bangladesh versus clinical COVID-19 surveillance: a longitudinal environmental surveillance study (December, 2019–December, 2021)
Source: Lancet Microbe. 2023 Jun;4(6):e442–51. doi: 10.1016/S2666-5247(23)00010-1 (PMC10069819; doi:10.1016/S2666-5247(23)00010-1)

THE LANCET
Microbe

Supplementary appendix

This appendix formed part of the original submission and has been peer reviewed. We post it as supplied by the authors.

Supplement to: Rogawski McQuade ET, Blake IM, Brennhofer SA, et al. Real-time sewage surveillance for SARS-CoV-2 in Dhaka, Bangladesh versus clinical COVID-19 surveillance: a longitudinal environmental surveillance study (December, 2019–December, 2021). *Lancet Microbe* 2023; published online April 3. https://doi.org/10.1016/S2666-5247(23)00010-1.

Supplementary appendix

Real-time sewage surveillance for SARS-CoV-2 in Dhaka, Bangladesh versus clinical COVID-19 surveillance: a longitudinal environmental surveillance study (Dec 2019 – Dec 2021)

Elizabeth T Rogawski McQuade,* Isobel M Blake,* Stephanie A Brennhofer, Md Ohedul Islam, Syed Shahnewaj Siraj Sony, Tonima Rahman, Md Hamim Bhuiyan, Sabrina Karim Resha, Erin G Wettstone, Lauren Hughlett, Claire Reagan, Sarah E Elwood, Yoann Mira, Ayesha S Mahmud, Kawsar Hosan, Md Raihanul Hoque, Md Masud Alam, Mahbubur Rahman, Tahmina Shirin, Rashidul Haque, Mami Taniuchi

*co-first authors

Supplementary Material

Supplementary Methods 3

Table S1. Sequences of primers and probes. 3

Supplementary Methods References 6

Supplementary Results 7

Figure S1. Visual depictions of the 8 wards before and after blue line tracing in Dhaka, Bangladesh. 8

Figure S2. Visual depictions of the 37 catchment sites throughout 8 wards in Dhaka, Bangladesh. 15

Figure S3. Weekly number of COVID-19 cases and log10 N1 copies per liter of sewage by week of SARS-CoV-2 test and environmental surveillance sample collection from Wards 8, 9, and 10 in the study area. 22

Figure S4. Weekly number of COVID-19 cases and log10 N1 copies per liter of sewage by week of SARS-CoV-2 test and environmental surveillance sample collection from Wards 2, 3, 5, 18 and 19 in the study area. 23

Figure S5. The correlation between the previous day's and the mean of the previous 3 days' of rainfall data and log10 copies of 1:100 diluted HF183 (A, B) and CrAssphage (C, D) viral load from March – July 2021. 24

Table S2. Sample assessment of the availability of clinical testing at the ward level at two different one week time periods throughout the pandemic. 25

Figure S6. The correlation between sewage viral load and logged case data from July 2020 – December 2021 by weekly lag in the study area. 26

Figure S7. The correlation between sewage viral load and logged case data from July 2020 – December 2020 by weekly lag in the study area. 27

Figures S8. The correlation between sewage viral load and logged case data from January 2021 – June 2021 by weekly lag in the study area. 28

Figure S9. The correlation between sewage viral load and logged case data from July 2021 – December 2021 by weekly lag in the study area. 29

Table S3. Comparing the correlation between case data and SARS−CoV−2 viral load in environmental surveillance in the study area. 30

Figure S10. Cross-correlations comparing the correlation between COVID-19 clinical case data and SARS-CoV-2 viral load in environmental surveillance data from July 2020-December 2021 from the study area. 31

Supplementary Methods

Site development

Once the sewage lines were mapped and digitized to shapefiles , Novel-T, a mapping company, built interactive maps for our study area using the sewer line shapefiles, WorldPop data,[1] 2m resolution DTM maps (AW3D, Tokyo, Japan), and digital elevation models at 2m resolution.[2] We identified prospective environmental surveillance (ES) based on the catchment area coverage and catchment population using the Watershed Tool and the Population Selector Tool built into the interactive maps. We then selected the ES sites if the site was accessible throughout the year, away from industrial wastes, and if the wastewater had a pH of around 7 and high total dissolved solids (>250 mg/L).

Blue line tracing and digitization of informal and formal sewage lines

For blue line tracing, multiple field teams went to the field sites to walk and cover every km of the study area. They traced all the informal and formal sewage lines and marked the flow direction on a physical map. At the end of each day, these lines were manually digitized using QGIS, a free and open-source Geographic Information System (https://www.qgis.org/en/site/index.html), to create shapefiles (appendix).

Environmental sample collection

Aquaread probe
Sensors detected and recorded conductivity, date, depth, dissolved oxygen, GPS, pH, oxidation reduction potential, resistivity, salinity, sea water specific gravity, temperature, time, total dissolved solids, and turbidity.

BMFS grab sample collection and processing
Six-litre grab samples of wastewater are collected using the collection bag, sealed, and placed in clean buckets for transportation back to a field office. Then, we filtered the sample through ViroCap filters at the field office until the 6L went through the filter or when 45 min has elapsed. Subsequently, the ViroCap filter housing was then placed on ice packs to maintain cold chain during transport to the International Centre for Diarrhoeal Disease Research, Bangladesh (icddr,b) laboratory for further processing

Virus elution, concentration, and total nucleic acid extraction
The virus was eluted using 1.5% beef extract, 0.05 M glycine, pH 9.5 eluent solution and further concentrated using skim milk flocculation.[3,4] Total nucleic acid (TNA) was extracted from the skim milk pellet using the QIAamp Stool Mini Kit (Qiagen, Gaithersburg, MD, USA) with a slightly modified manufacturer's protocol.[18] An extraction blank was included per batch of extraction to monitor for contamination. The TNA was stored in -80°C until further testing.

RT-qPCR for SARS-CoV-2
The PCR primers were designed to amplify the virus nucleocapsid (N) gene region of the SARS-CoV-2 genome. The PCR probes were designed to capture specific amplicon(s). The primers and probes were designed by the Center for Disease Control and Prevention (sequences of the primers and probes are shown in Table S1.[5] The primer probe mixes for N1 and N2 amplicons were part of the 2019-nCoV CDC EUA Kit (Integrated DNA Technologies, Inc., Coralville, IA).

Table S1. Sequences of primers and probes.

Target	Forward primer (5′→3′)	Reverse Primer (5′→3′)	Probe (5′→3′)
N1	GAC CCC AAA ATC AGC GAA AT	TCT GGT TAC TGC CAG TTG AAT CTG	FAM-ACC CCG CAT /ZEN/ TAC GTT TGG TGG ACC-3IABkFQ
N2	TTA CAA ACA TTG GCC GCA AA	GCG CGA CAT TCC GAA GAA	FAM-ACA ATT TGC /ZEN/ CCC CAG CGC TTC AG-3IABkF

Each 20-µl RT-qPCR for N1 included 5 µl of 4X qScript XLT One-Step RT-qPCR Toughmix (Quanta Biosciences, Beverly, MA), 1.5 µl of primer probe mix N1 (Integrated DNA Technologies, Inc., Coralville, IA), 8.5 µl of nuclease free water, and 5 µl of total nucleic acid or positive control or nuclease free water (no template control). Each 20-µl RT-qPCR for N2 included 5 µl of 4X qScript XLT One-Step RT-qPCR Toughmix (Quanta Biosciences, Beverly, MA, USA), 1.5 µl of primer probe mix N2 (Integrated DNA Technologies, Inc., Coralville, IA), 8.5 µl of nuclease free water, and 5 µl of total nucleic acid or positive control or nuclease free water (no template control). 2019-nCoV-N Positive Control (Integrated DNA Technologies, Inc., Coralville, IA), a plasmid positive control, was diluted to a final working concentration of 200 copies/µL and included as a positive control template for every PCR plate while nuclease free water was included as a no template control (negative control). Cycling conditions included 10 min of reverse transcriptase enzyme activation and cDNA synthesis at 50°C, 3 min of initial denaturation and enzyme activation at 95°C, and 40 cycles of 5 s at 95°C and 30 s at 60°C. The PCR was performed on the CFX96 (Bio-Rad, Hercules, CA) and the results were analysed with CFX Maestro software v1.1 (Bio-Rad, Hercules, CA). Threshold cycles (Cts) were determined after setting the baseline threshold.

Analytical performance of real-time RT-qPCR N1 and N2 gene assays

Standard curves for the N1 and N2 gene assays were generated by testing 10-fold serial dilutions of the SARS-CoV-2 RUO Plasmid Controls (IDT, Coralville, IA) in triplicate (concentration ranged from 200,000 to 0.2 copies/µL). Serial dilutions of SARS-CoV-2 RUO Plasmid Controls (IDT, Coralville, IA) showed that the limit of detection (defined as the lowest dilution where 10/10 samples were detected) was the same for N1 and N2 (10 copies/µl). The PCR efficiencies calculated from the slopes of the standard curves were 98% and 103% for the N1 and N2 assays, respectively

qPCR for faecal indicator organisms HF183 and CrAssphage

Each assay was tested in 25 µl reaction which included 12.5 µl of 2x TaqMan Environmental master mix (Thermo Fisher Scientific, Inc., Waltham, MA), 0.25 µl of 100 µM forward and reverse primers (final concentration 1 µM), 0.02 µl of 100 µM probe (final concentration 0.08 µM), 6.98 µl nuclease free water, and 5 µl total nucleic acid template. The synthetic fragment of HF183 and CrAssphage (Integrated DNA Technologies, Inc., Coralville, IA) and nuclease free water were included in every plate as positive and no-template control respectively. The qPCR was performed on CFX96 (Bio-Rad, Hercules, CA) which cycling condition included initial denaturation at 95°C for 10 min, followed by 40 cycles of denaturation at 95°C for 15s and annealing/extension at 60˚C for 1 min.

We tested a total of 396 samples for HF183 and CrAssphage. Out of these 396 samples, n=322 samples were positive for SARS-CoV-2. Of these samples, 100% (322/322) and 99.38% (320/322) were positive for CrAssphage and HF183, respectively.

Statistical analysis

Human faecal indicators, rainfall, and catchment area population size

We assessed the correlation between the mean $\log_{10}$ viral load of CrAssphage and HF183 in wastewater with $\log_{10}$ mean of the catchment area population size (scaled to per 1000 persons) from four weeks in March 2021, May 2021, and July 2021 to determine if it was possible to normalize measured concentrations of SARS-CoV-2 to the population contributing to each sewage sample. The months were chosen to ensure data was collected during the dry (March) and rainy (July) seasons. We proceeded with 1:100 dilutions of HF183 and CrAssphage as they were detected in every sample and had low Ct values. We assessed the correlation via linear regression between the mean $\log_{10}$ viral load of HF183 and CrAssphage in wastewater and rainfall (mm) [6] from four weeks in March 2021, May 2021, and July 2021. We used linear regression models to examine the correlations at the

site, ward, and overall levels with the previous one days and the mean of the previous three days' of rainfall, adjusting for site using fixed effects.

Analysis of case data and correlation with sewage data

We compared the environmental samples on the $\log_{10}$ scale to the clinical case data on the normal and $\log_{10}$ scales via the Shapiro-Wilk normality test. The logged case data had a normal distribution (p=0.10) while the unlogged case data did not (p<0.001). Therefore, we used logged clinical case data. Next, we assessed the Pearson correlation between the viral load of environmental samples and the logged clinical case data from the study region where each week of environmental sample data was compared against clinical case data from the same week (0 lag), the next week (1-week lag), and 2 weeks out (2-week lag) for the entire study and in six-month time periods (July 2020-December 2020, January 2021-June 2021, July 2021-December 2021). Case data prior to July 2020 was excluded due to inadequate testing prior to this time. Additionally, we estimated the cross-correlation between sewage viral load and clinical cases in the study region from July 2020-December 2021 via a linear model with generalized estimating equations adjusting for ward and temporal autocorrelation.[7] Logged case data was smoothed using a 7-day running average where we averaged the number of cases three days before, the day of, and three days after to account for a lack of or reduction in laboratory testing on weekends. We then estimated the cross-correlations for the entire study period and in six-month time periods (July 2020-December 2020, January 2021-June 2021, July 2021-December 2021) to examine if there were changes in the association between the logged clinical cases and viral load in the ES samples throughout the pandemic.

Supplementary Methods References

1. Jubayer MF, Kayshar MS, Al Emran M, Uddin MN, Alam Soeb MJ. Response to coronavirus disease 2019: Case study of one baking industry in Dhaka, Bangladesh. *Journal of Agriculture and Food Research*. 2020;2:100077. doi:10.1016/j.jafr.2020.100077

2. Environmental Sites. Accessed April 26, 2022. https://es.world/country/BGD/Dhaka

3. Development and Validation of the Skimmed Milk Pellet Extraction Protocol for SARS-CoV-2 Wastewater Surveillance | SpringerLink. Accessed April 11, 2022. https://link.springer.com/article/10.1007/s12560-022-09512-5

4. Abraham D, Mohan VR, Kang G. Skimmed Milk Flocculation Technique for Waste Water. protocols.io. Published May 27, 2021. Accessed April 26, 2022. https://www.protocols.io/view/skimmed-milk-flocculation-technique-for-waste-wate-buzmnx46

5. Centers for Disease Control and Prevention. Research Use Only 2019-Novel Coronavirus (2019-nCoV) Real-time RT-PCR Primers and Probes. Published February 11, 2020. Accessed April 11, 2022. https://www.cdc.gov/coronavirus/2019-ncov/lab/rt-pcr-panel-primer-probes.html

6. Buy Historical Weather Data for worldwide locations. Published 2021. https://www.worldweatheronline.com/hwd/shop/default.aspx

7. Højsgaard S, Halekoh U, Yan J. The R Package geepack for Generalized Estimating Equations. *Journal of Statistical Software*. 2006;15:1-11. doi:10.18637/jss.v015.i02

Supplementary Results

Analytical performance of real-time RT-qPCR N1 and N2 gene assays

Standard curves for the N1 and N2 gene assays were generated by testing 10-fold serial dilutions of the SARS-CoV-2 RUO Plasmid Controls (IDT, Coralville, IA) in triplicate (concentration ranged from 200,000 to 0.2 copies/µL). Serial dilutions of SARS-CoV-2 RUO Plasmid Controls (IDT, Coralville, IA) showed that the limit of detection (defined as the lowest dilution where 10/10 samples were detected) was the same for N1 and N2 (10 copies/µl). The PCR efficiencies calculated from the slopes of the standard curves were 98% and 103% for the N1 and N2 assays, respectively.

Trends in environmental surveillance and clinical case data

We tested a total of 396 samples for HF183 and CrAssphage. Out of these 396 samples, n=322 samples were positive for SARS-CoV-2. Of these samples, 100% (322/322) and 99.38% (320/322) were positive for CrAssphage and HF183, respectively.

There was no correlation between catchment area population size and concentrations of 1:100 dilutions of HF183 (*R*= 0.02 (March 2021); *R*= 0.12 (May 2021); *R*= -0.09 (July 2021)) or 1:100 dilutions of CrAssphage HF183 (*R*= 0.11 (March 2021); *R*= 0.16 (May 2021); *R*= -0.10 (July 2021)) at any time point. There were no associations between concentrations of 1:100 dilutions of HF183 and rainfall by individual sites or by wards (adjusted and not adjusted for site). The overall adjusted model indicated there was a 0.01 decrease in $\log_{10}$ copies of 1:100 diluted HF183 viral load for every 1mm increase in rainfall in the previous day (Mean difference: -0.01; 95% CI: -0.02, -0.00). A similar association was observed with rainfall in the previous three days (Mean difference: -0.01; 95% CI: -0.03, -0.00). There were no associations between rainfall and concentrations of 1:100 dilutions of CrAssphage by wards (adjusted and non-adjusted for site) or overall (adjusted and non-adjusted for site) (Figure S5). There was one significant association in Ward 19, Site 10, however, the association was positive (Mean difference: 0.01; 95% CI: 0.00, 0.02), which suggested that CrAssphage concentration increased as rainfall increased.

Figure S1. Visual depictions of the 8 wards before and after blue line tracing in Dhaka, Bangladesh. Red lines indicate ward boundaries. Green lines indicate formal sewage lines. Blue lines indicate informal sewage lines that were blue line traced. Areas not blue line traced within the wards include: official, restricted, non-residential, under construction areas.

Ward 2: Before

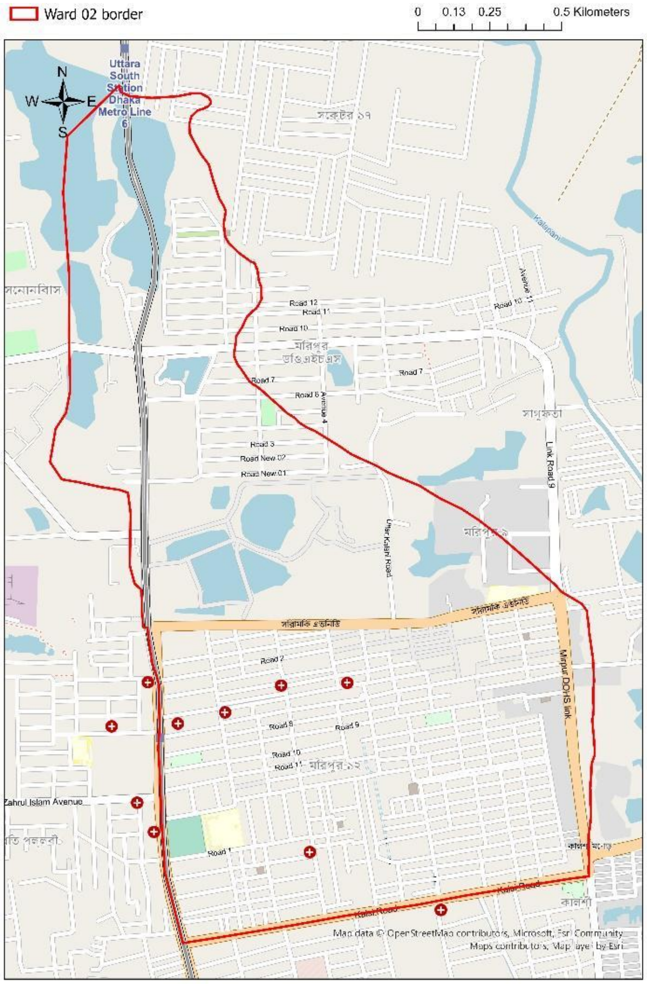

Ward 2: After

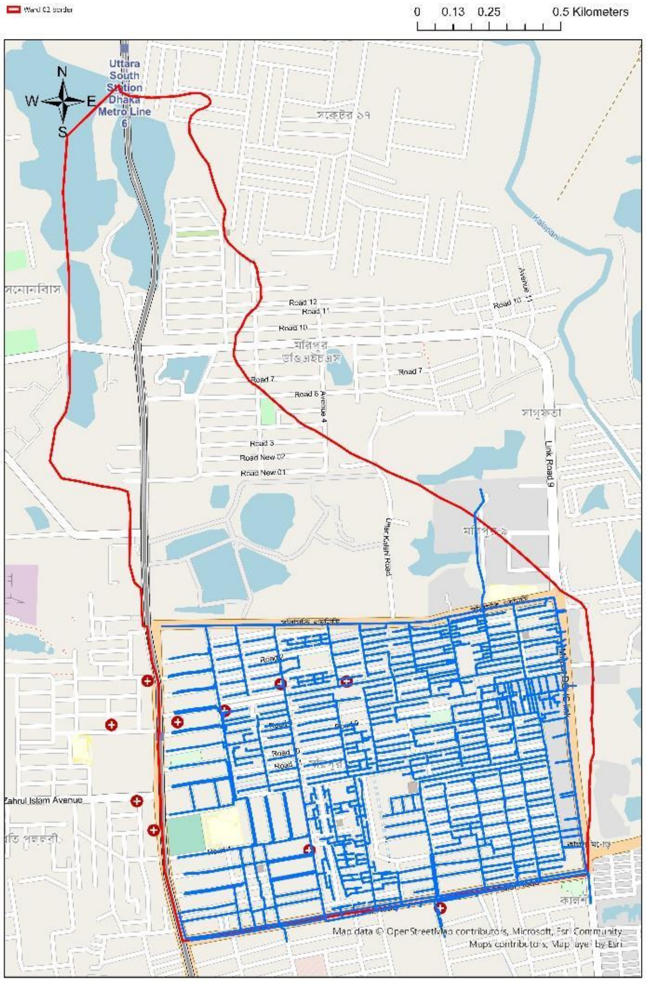

Ward 3: Before

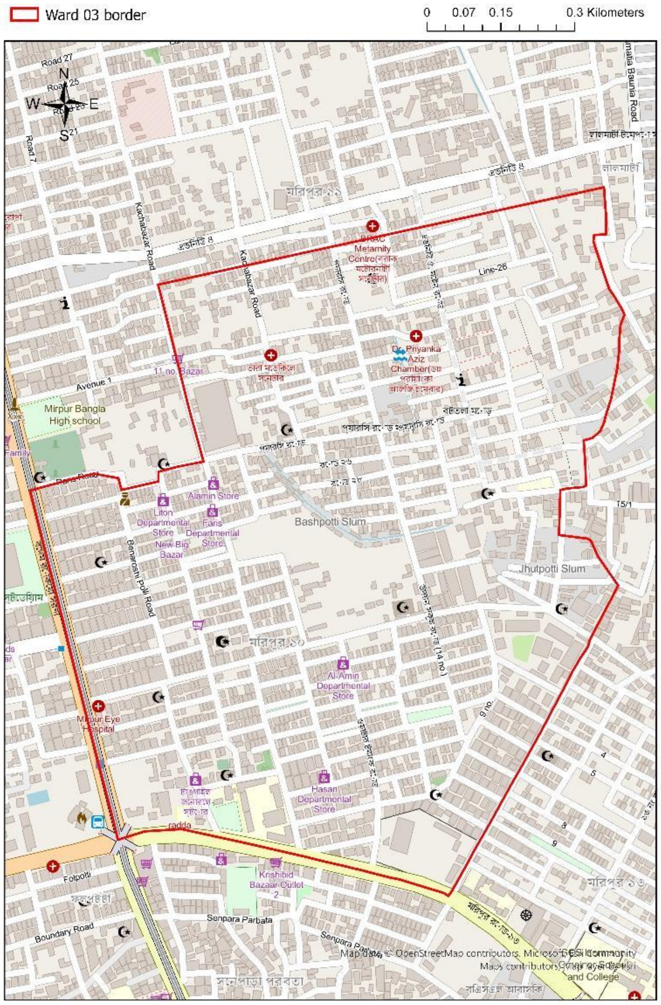

Ward 3: After

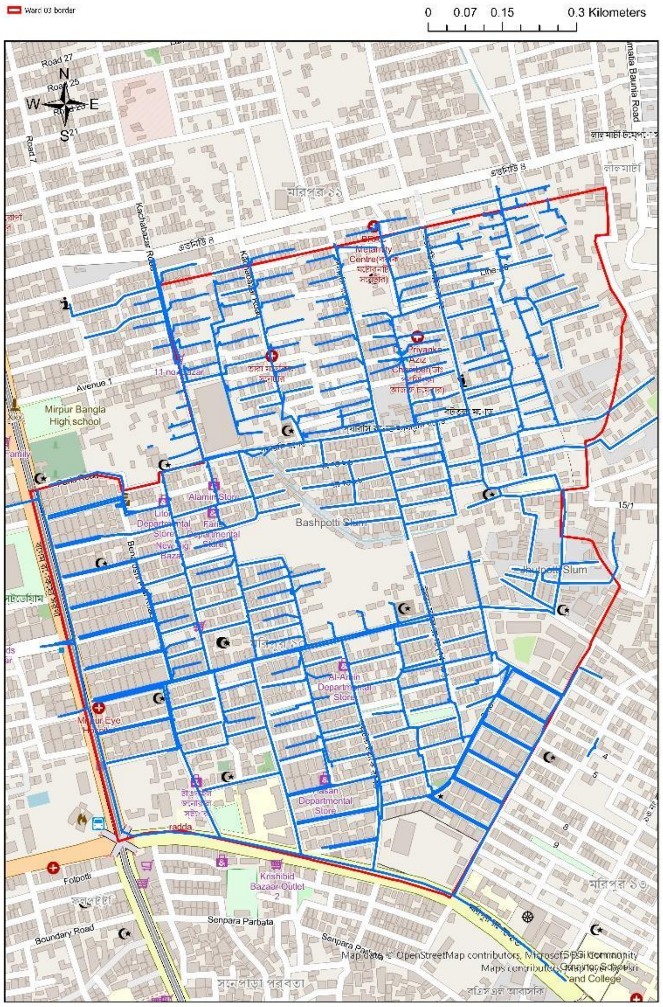

Ward 5: Before

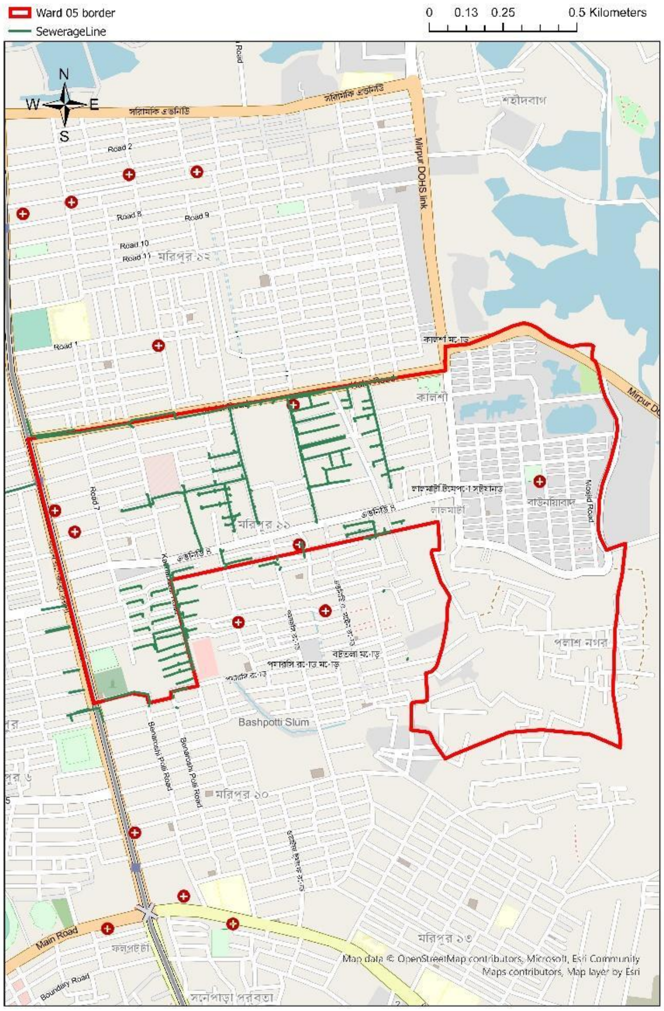

Ward 5: After

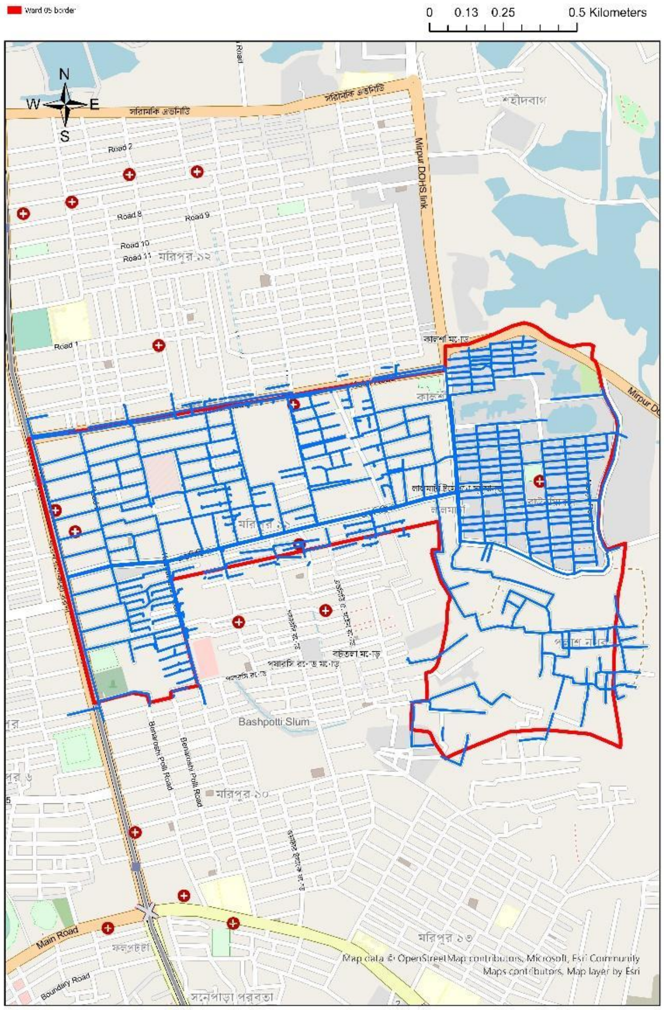

Ward 8: Before

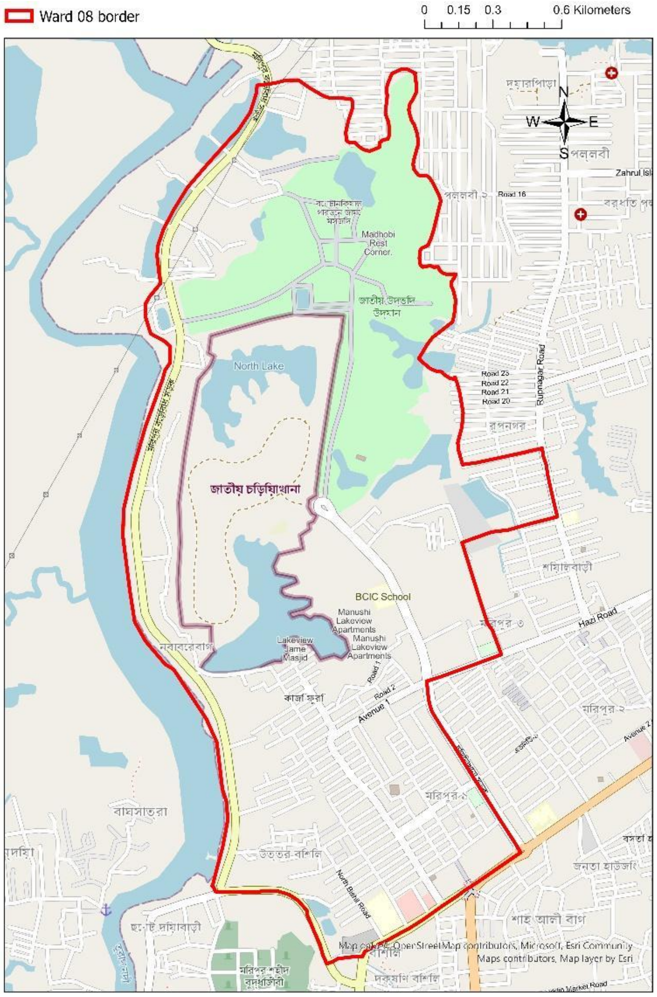

Ward 8: After

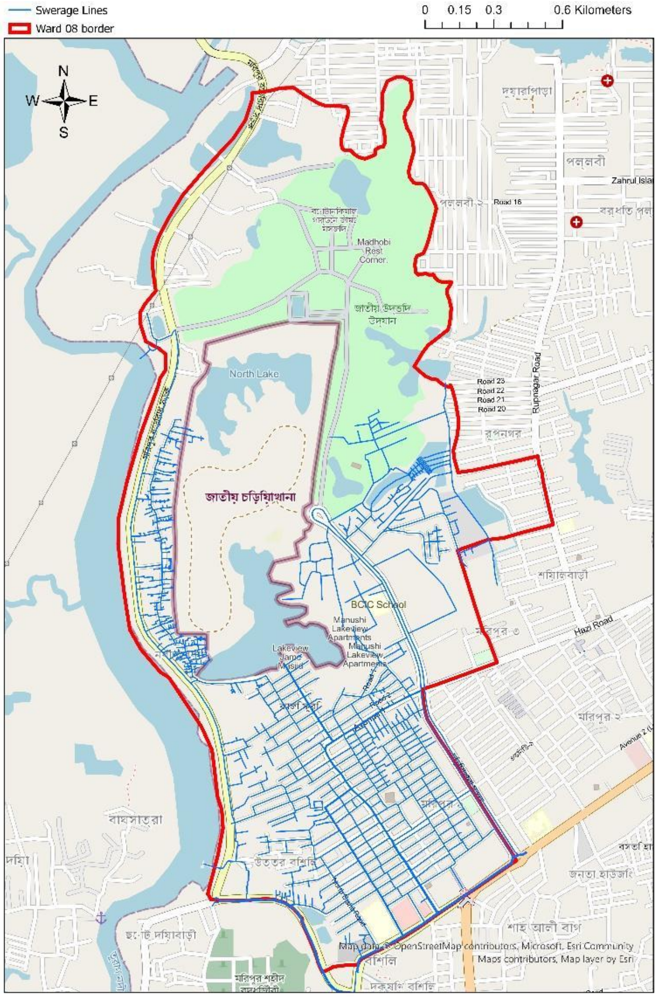

Ward 9: Before

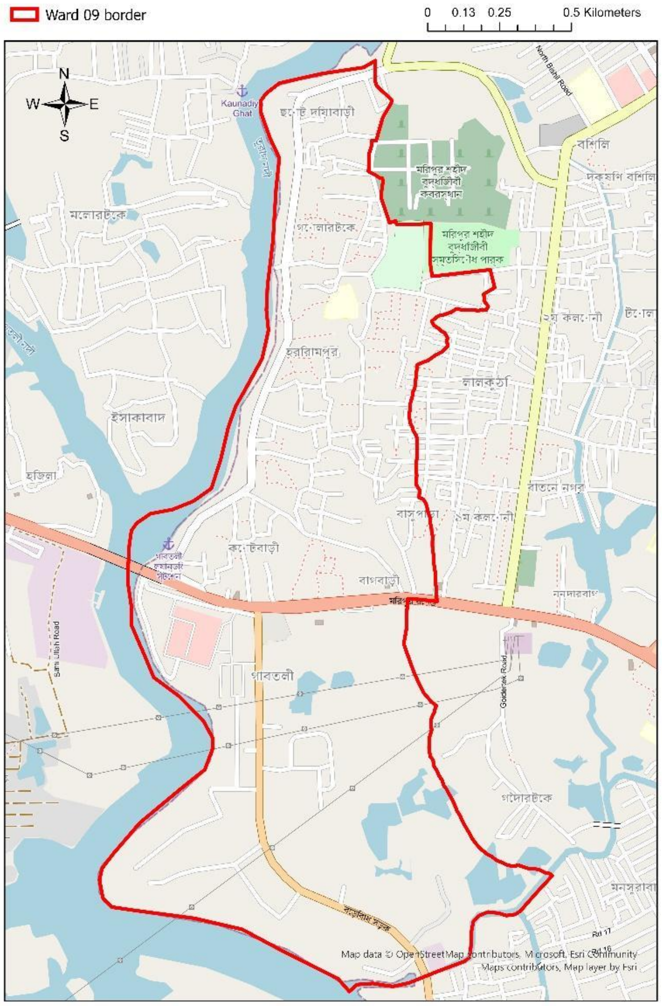

Ward 9: After

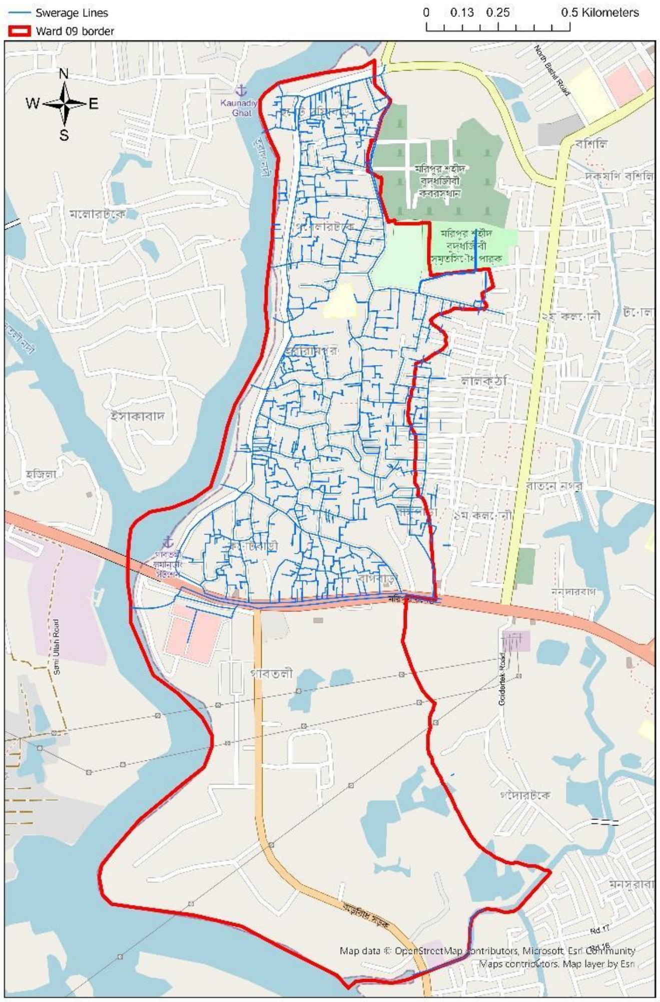

Ward 10: Before

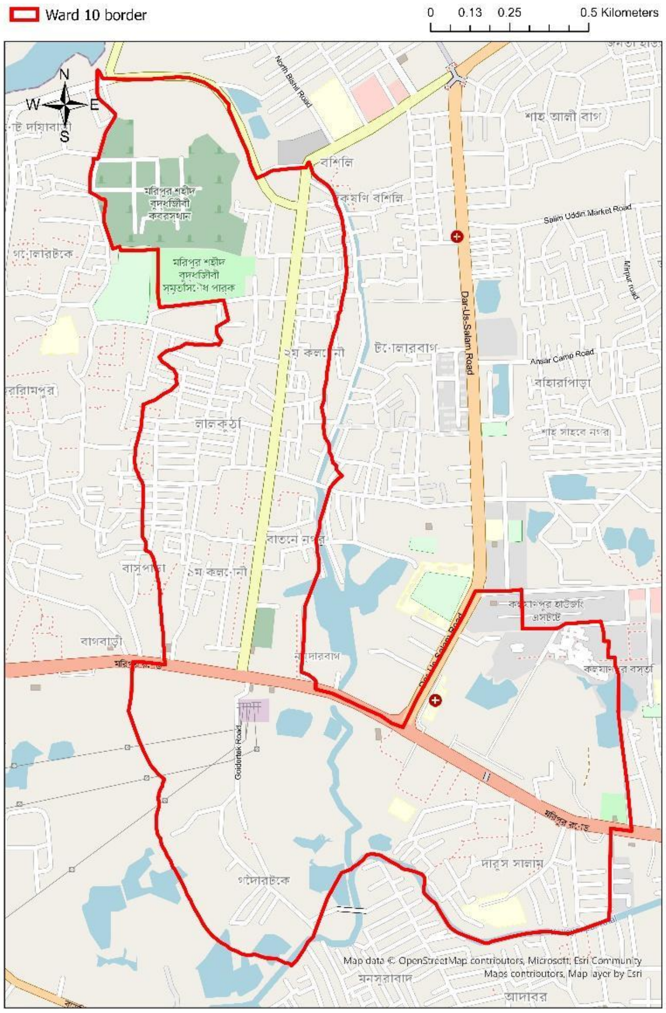

Ward 10: After

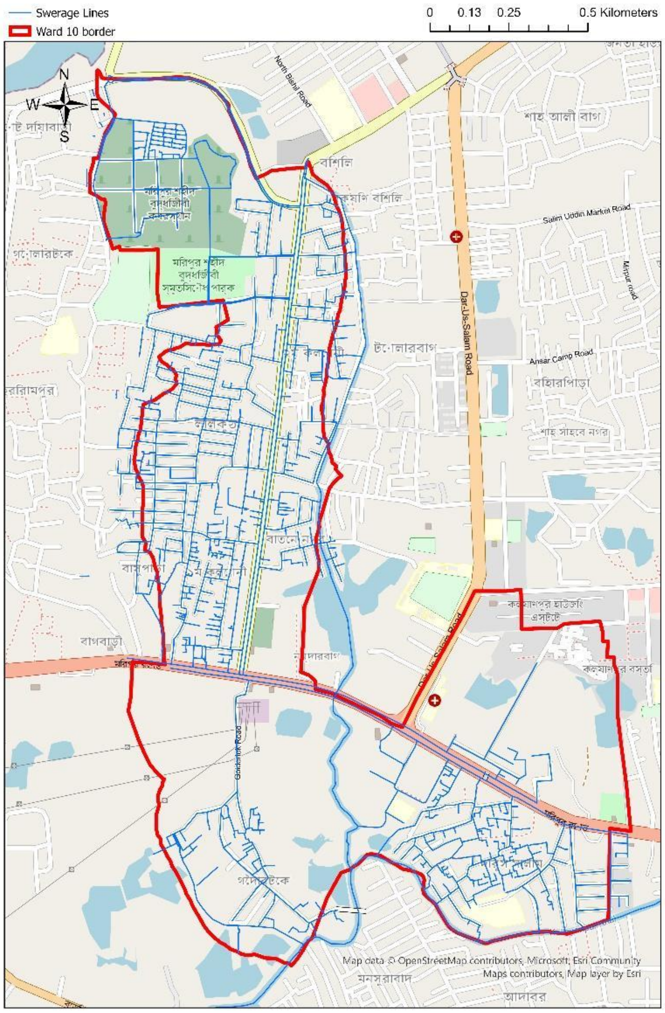

Wards 18 and 19: Before

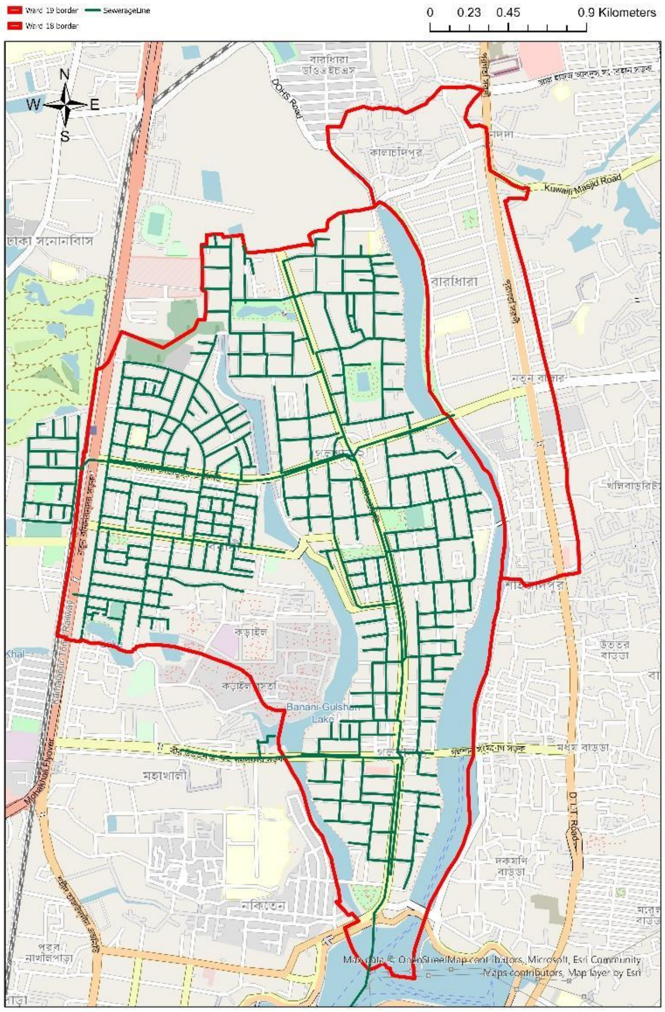

Wards 18 and 19: After

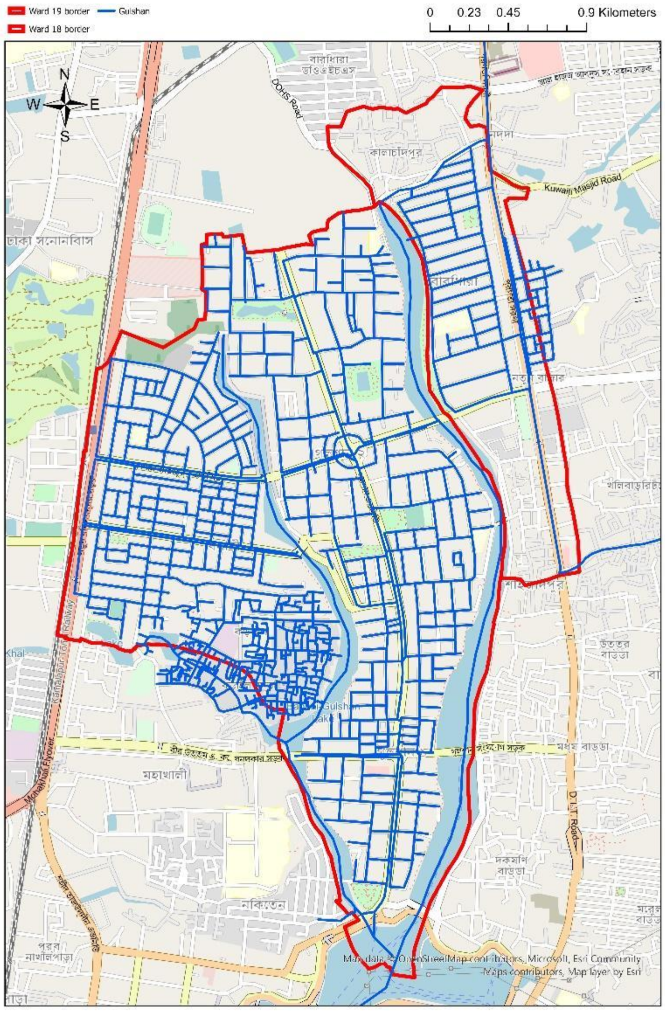

Figure S2. Visual depictions of the 37 catchment sites throughout 8 wards in Dhaka, Bangladesh.

Ward 2 Site 01
Pollobi Women Degree College, Kalshi

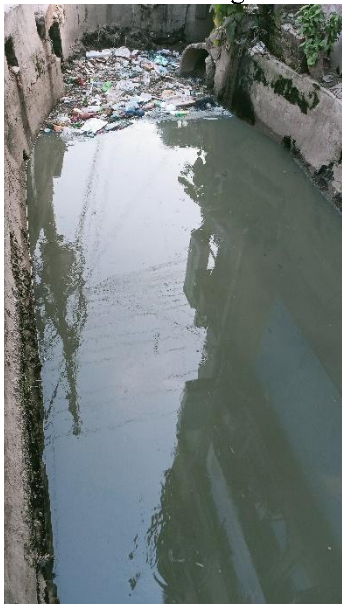

Ward 8 Site 04
Muktijoddha Complex Manhole

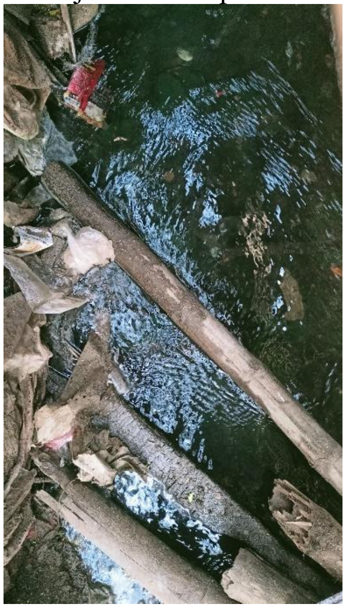

Ward 18 Site 02
Suvastu Shopping Complex, Badda

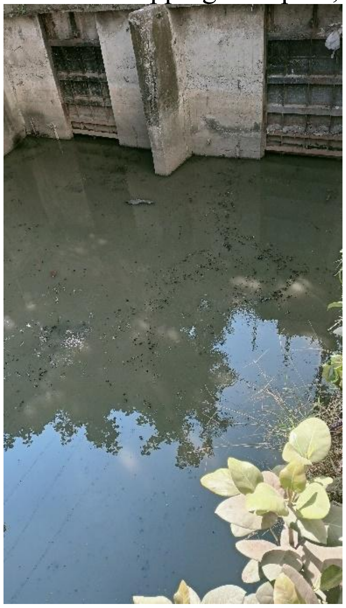

Ward 2 Site 02
Sagufta

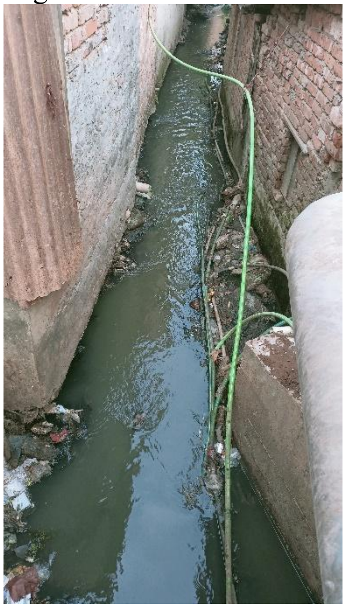

Ward 8 Site 05
Mazar Road Corner Slab

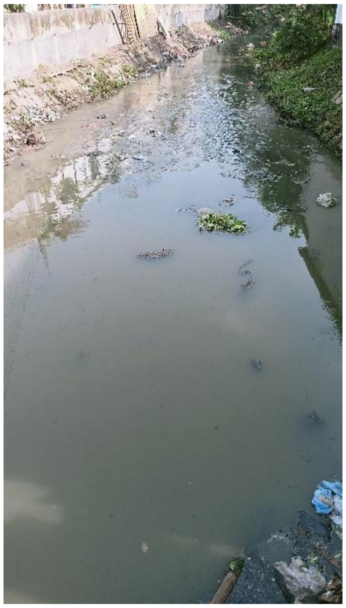

Ward 19 Site 01
Brac Center, Gulshan

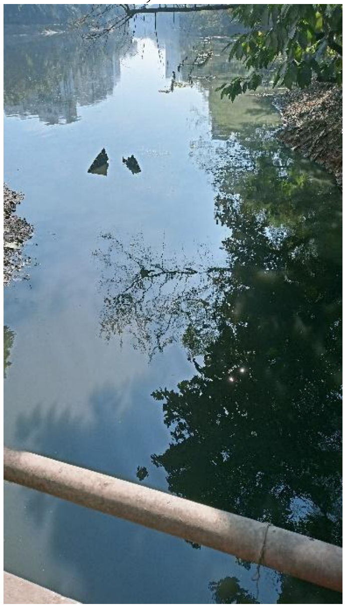

Ward 3 Site 01
Paris Road Khal

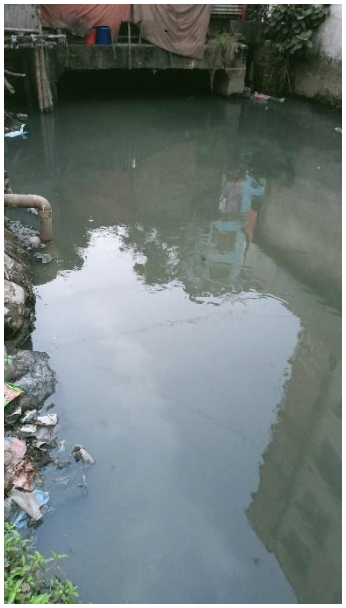

Ward 9 Site 01
Borobazar Para, Beribadh, Gabtoli

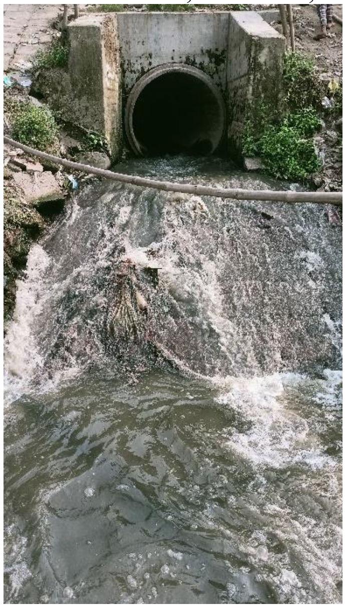

Ward 19 Site 02
Banani-11 Bridge-1

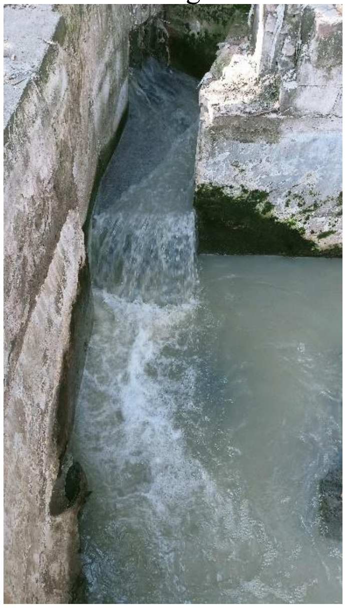

Ward 3 Site 03
Avenue-4 Culvert-2

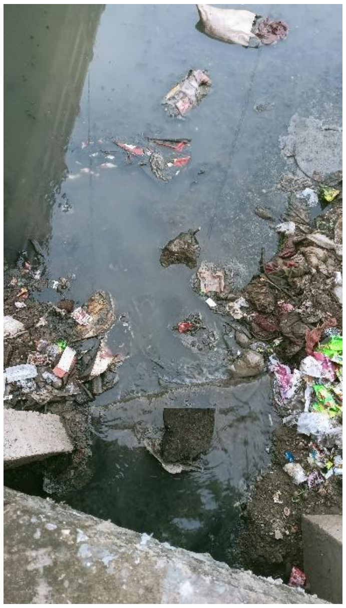

Ward 9 Site 02
Palpara Ghat, Beribadh Gabtoli

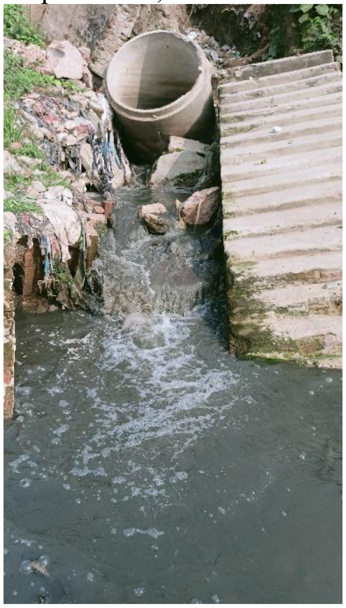

Ward 19 Site 03
Banani-11 Bridge-2

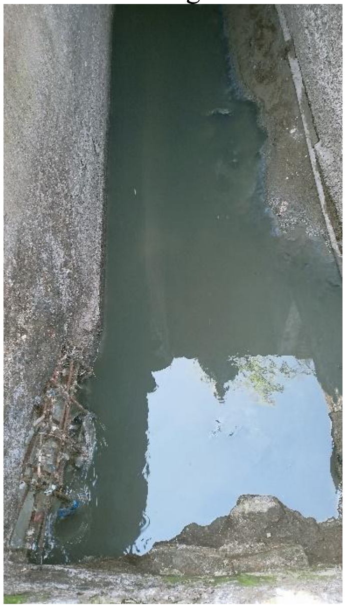

Ward 5 Site 01
Avenue-4 Culvert-1

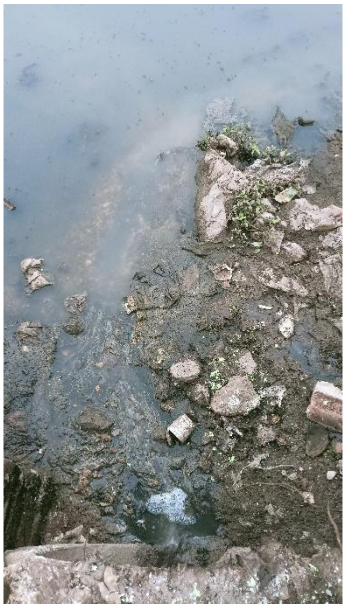

Ward 9 Site 03
Ananda Nagar Block-D

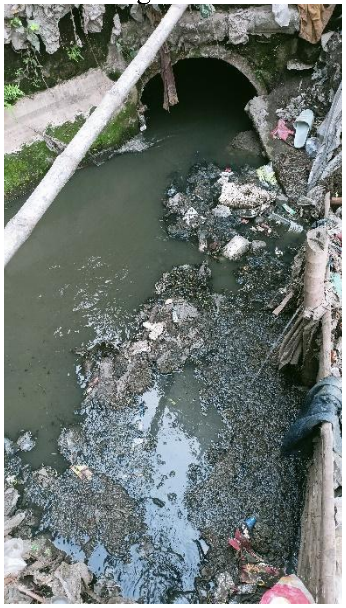

Ward 19 Site 04
Karail Bosti

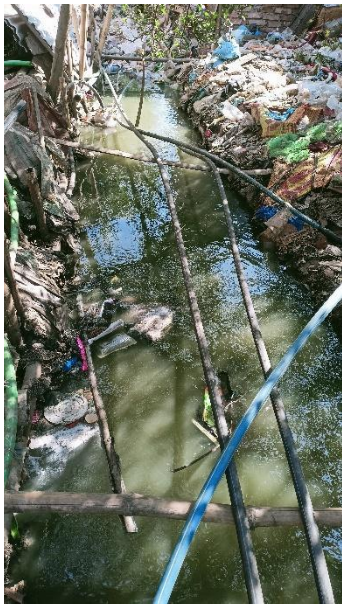

Ward 5 Site 03
22 Floor Garments

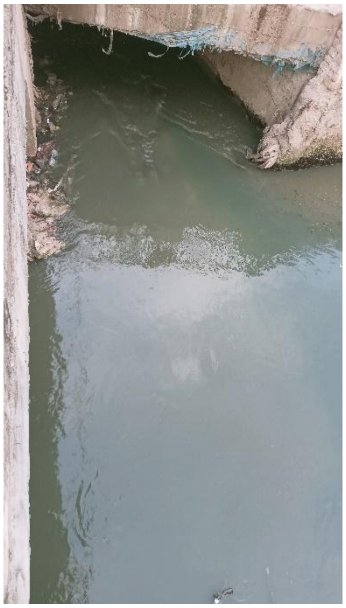

Ward 9 Site 04
Hanif Bus Counter, Gabtoli

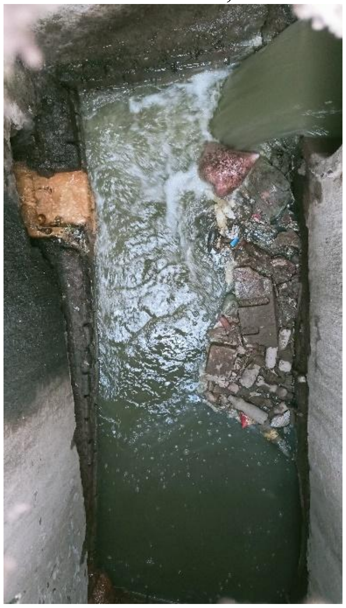

Ward 19 Site 05
Gulshan Niketon Link Bridge

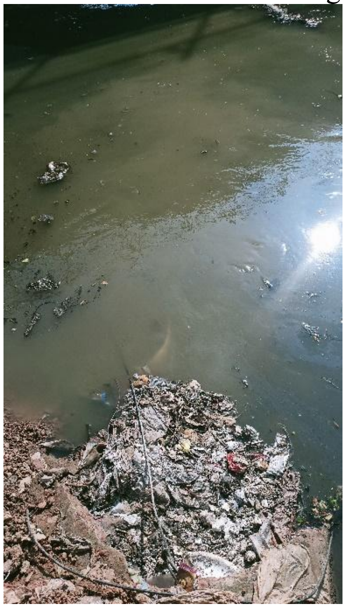

Ward 5 Site 04
Bauniabad Switch Gate Calvert

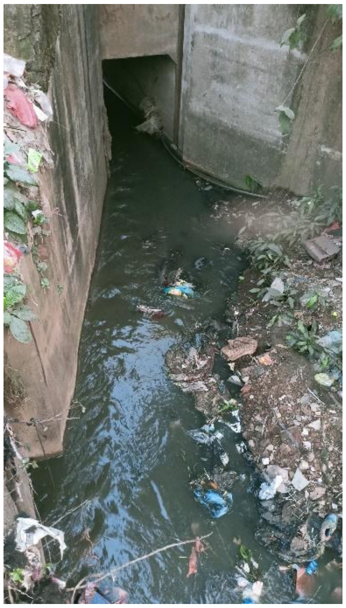

Ward 10 Site 01
Society Balur Math, Darus Salam

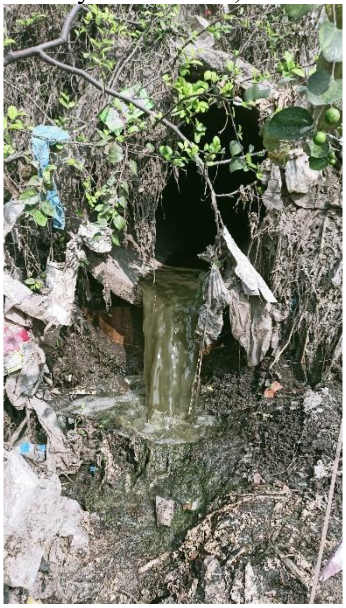

Ward 19 Site 06
Gulshan-2 Link Bridge-1

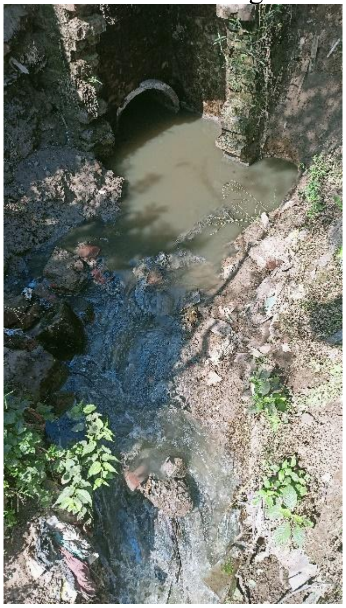

Ward 5 Site 05
22 Teki Khal

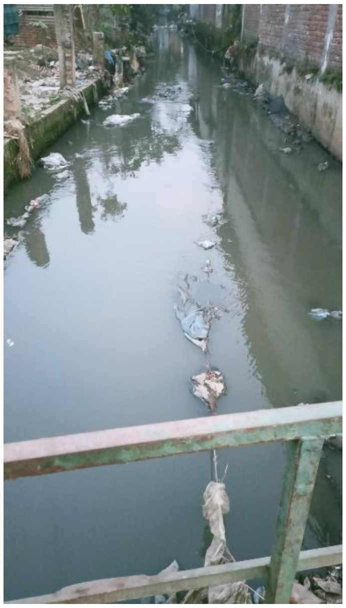

Ward 10 Site 02
Boat Stand, Darus Salam Road

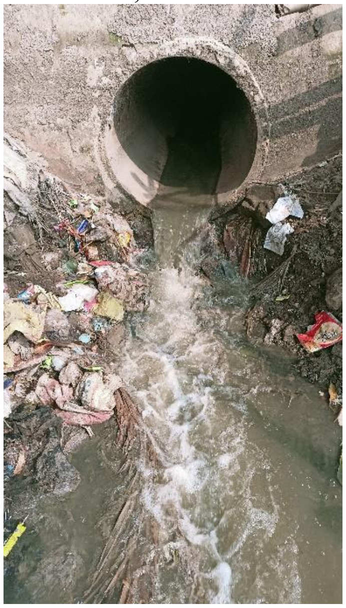

Ward 19 Site 07
Gulshan-2 Link Bridge-2

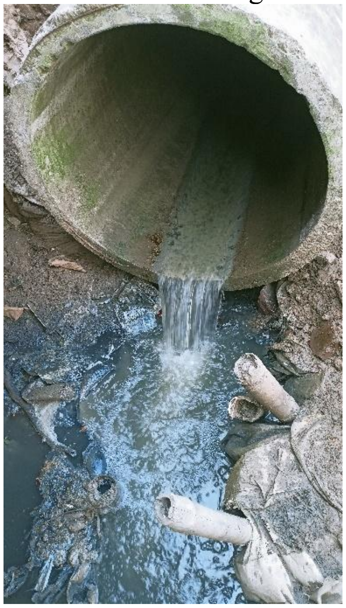

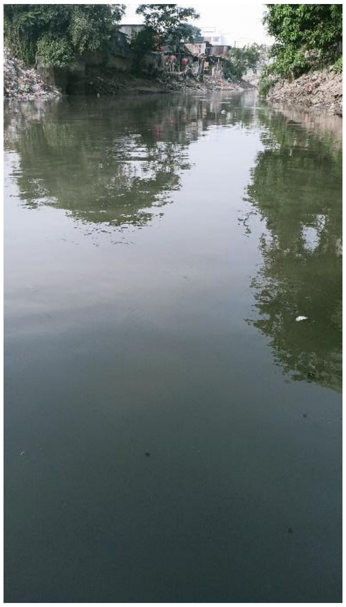

Ward 5 Site 06
Mirpur Dohs Link Road-2

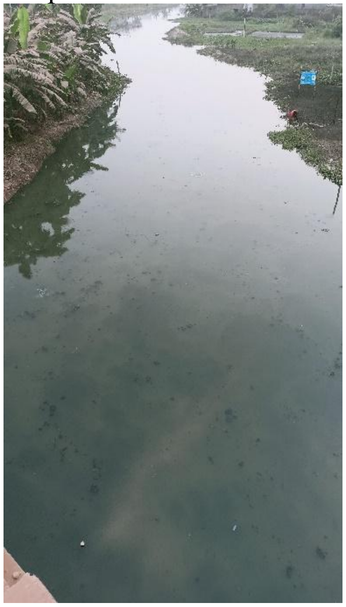

Ward 10 Site 03
Btenia Jame Masjid

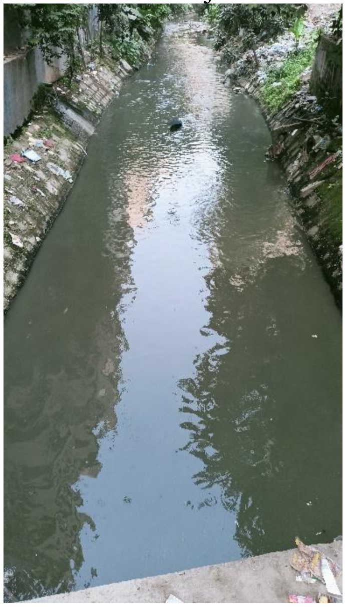

Ward 19 Site 08
Gulshan Circle-1 Manhole

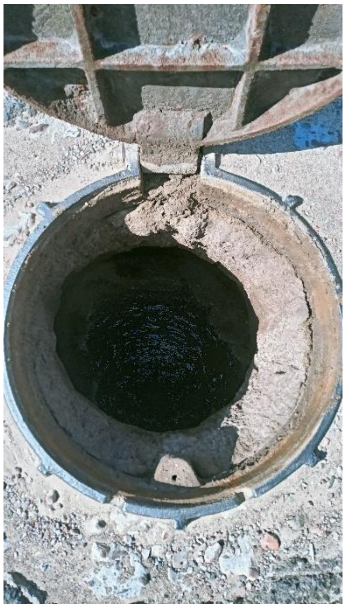

Ward 5 Site 07
Mirpur Dohs Link Road-1

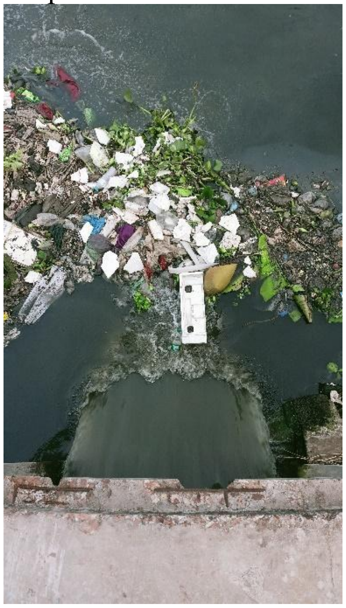

Ward 10 Site 04
Lalkuthir Bazar, Mirpur Mazar Road

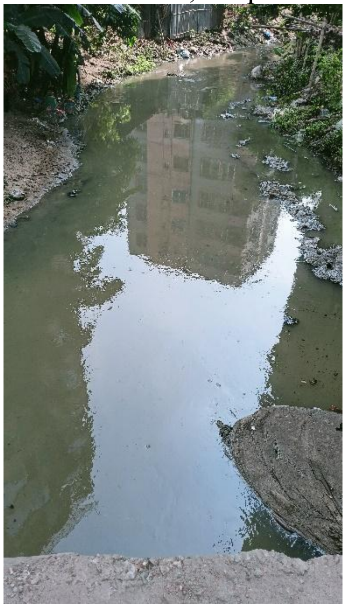

Ward 19 Site 09
Gulshan Circle-2 Manhole

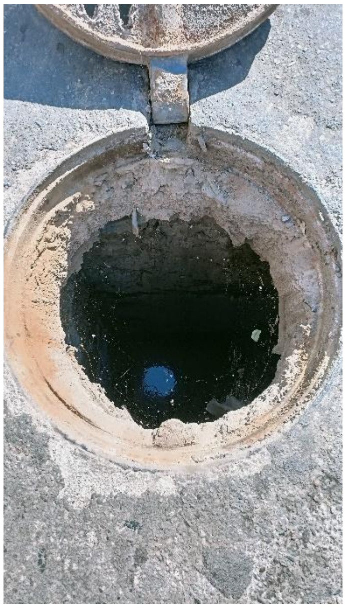

Ward 8 Site 01
Switch Gate, Beribadh

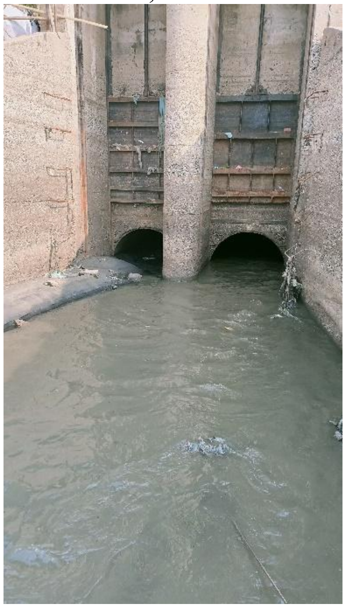

Ward 10 Site 05
Shahjadpur Bus Stand, Gabtoli

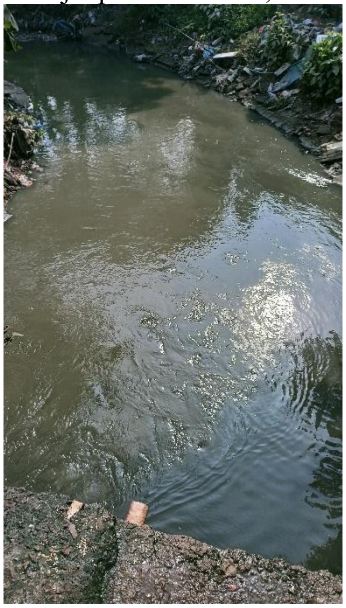

Ward 19 Site 10
Karail Bosti-2

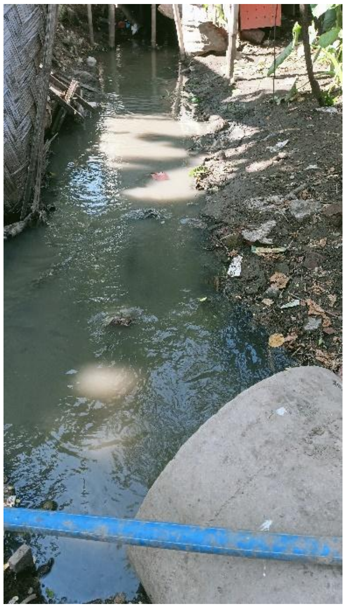

Ward 8 Site 02
Momen Dewan Bosti

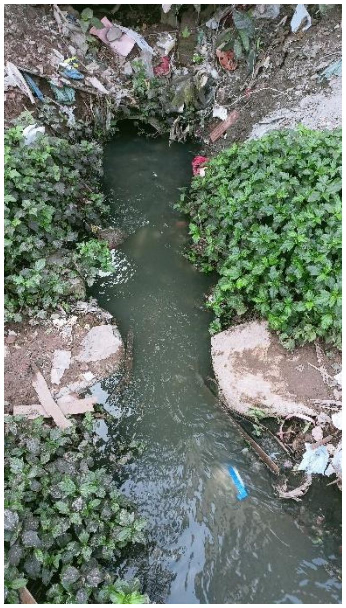

Ward 18 Site 01
Road-9 Baridhara Park

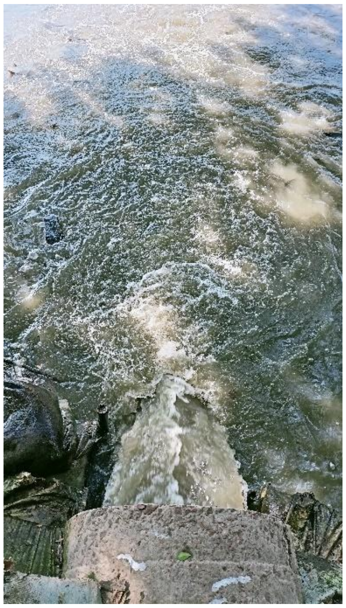

Ward 19 Site 11
Karail Bosti-3

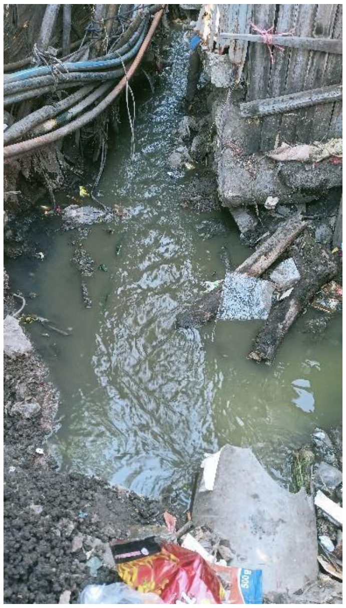

Ward 8 Site 03
Nobaberbagh Bus Stand Manhole

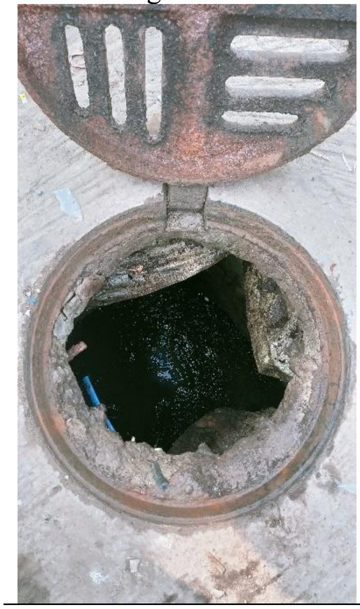

Figure S3. Weekly number of COVID-19 cases and log10 N1 copies per liter of sewage by week of SARS-CoV-2 test and environmental surveillance sample collection from Wards 8, 9, and 10 in the study area.

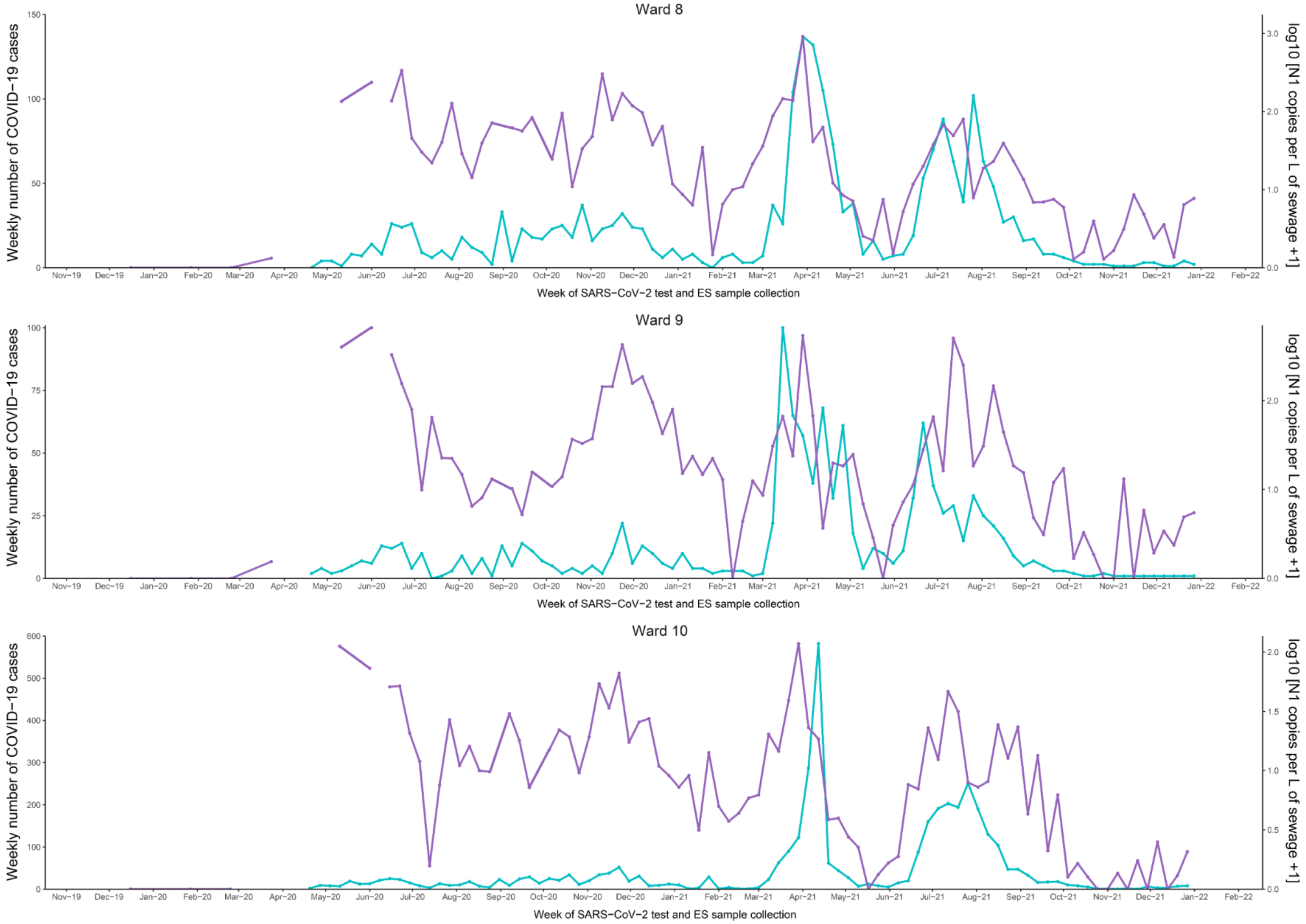

Figure S4. Weekly number of COVID-19 cases and log10 N1 copies per liter of sewage by week of SARS-CoV-2 test and environmental surveillance sample collection from Wards 2, 3, 5, 18 and 19 in the study area.

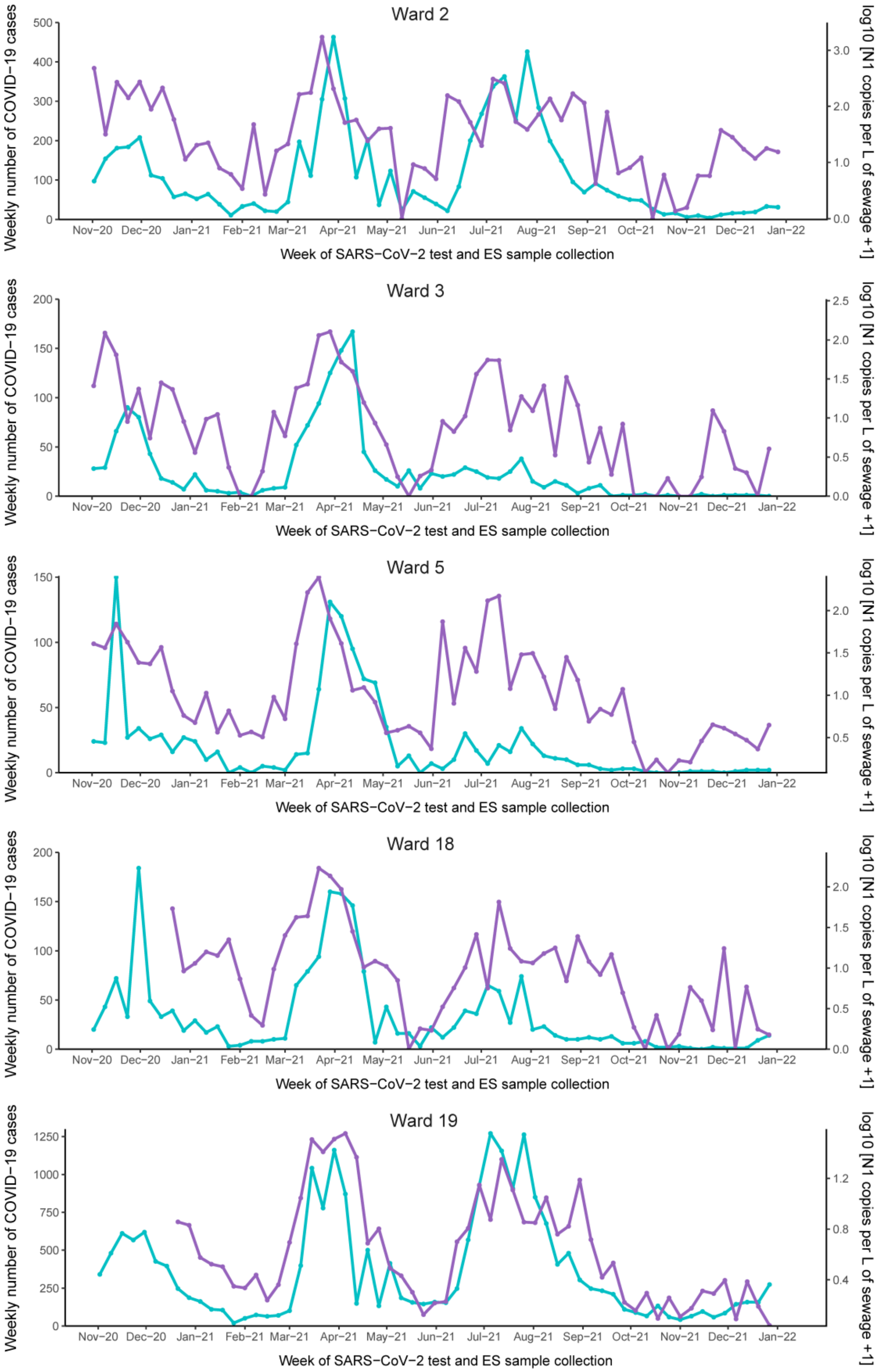

Figure S5. The correlation between the previous day's and the mean of the previous 3 days' of rainfall data and log10 copies of 1:100 diluted HF183 (A, B) and CrAssphage (C, D) viral load from March – July 2021. Each datapoint represents one sample from one site. Sample collection took place four days per week. The same rainfall data was attributed to all sites collected on the same day.

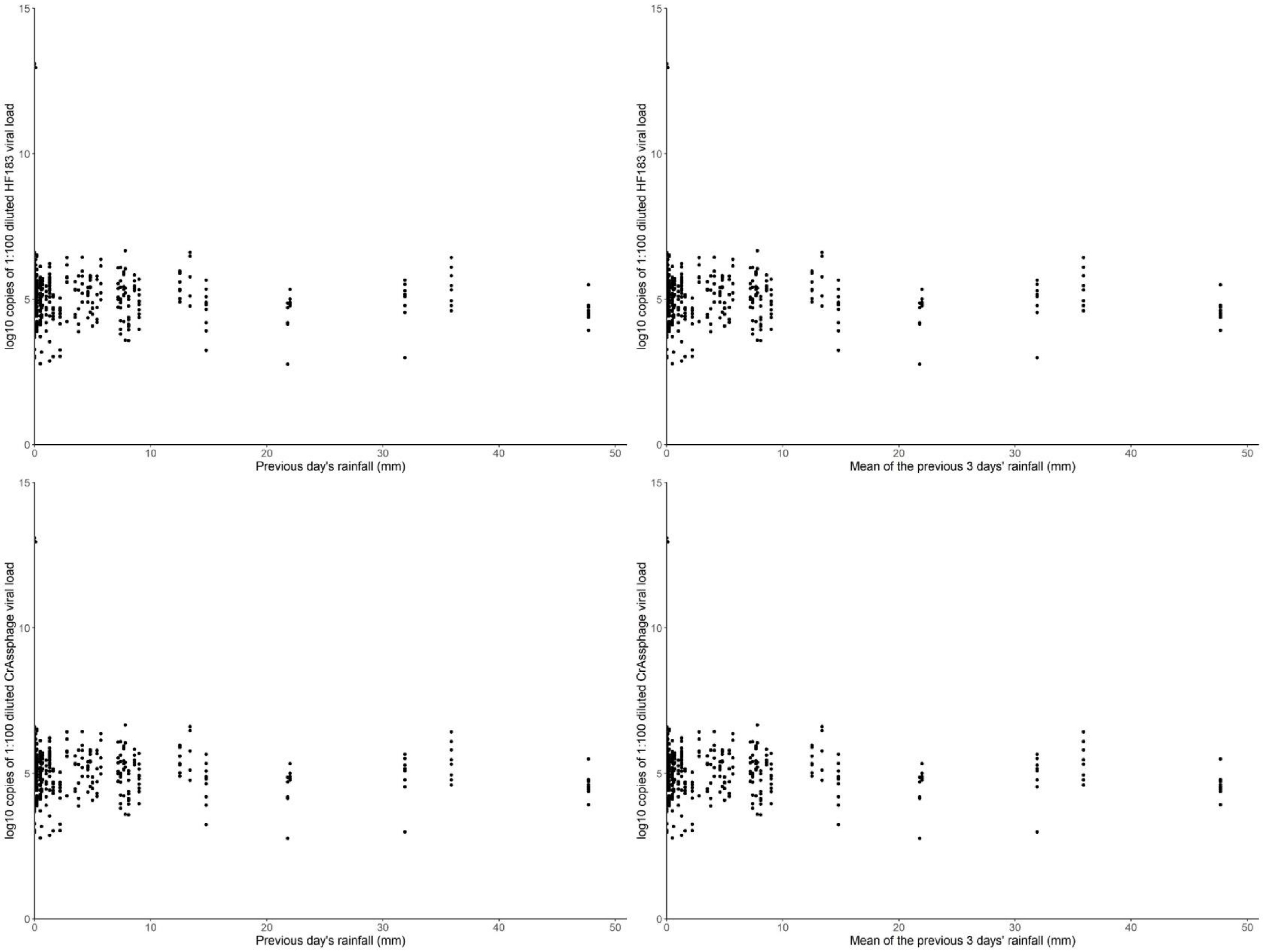

Table S2. Sample assessment of the availability of clinical testing at the ward level at two different one week time periods throughout the pandemic.

Ward	Population total	November 2020			November 2021[a]		
		Number of tests	Tests per 100,000 persons	Ratio to referent group	Number of tests	Tests per 100,000 persons	Ratio to referent group
02	117084	113	97	7	30	26	4
03	91474	173	189	15	17	19	3
05	113496	41	36	3	7	6	Referent
08	82474	44	53	4	15	18	3
09	53525	7	13	Referent	0	0	--
10	92585	117	126	10	7	8	1
18	41704	211	506	39	109	261	42
19	142413	2283	1603	123	611	429	70

The referent group was chosen based on the lowest non-zero number of tests per 100,000 individuals at each time point. Numbers were rounded to the nearest whole number. [a] Based on a random 20% sample.

Figure S6. The correlation between sewage viral load and logged case data from July 2020 – December 2021 by weekly lag in the study area. From top to bottom: 0 week, 1 week, and 2-week lag.

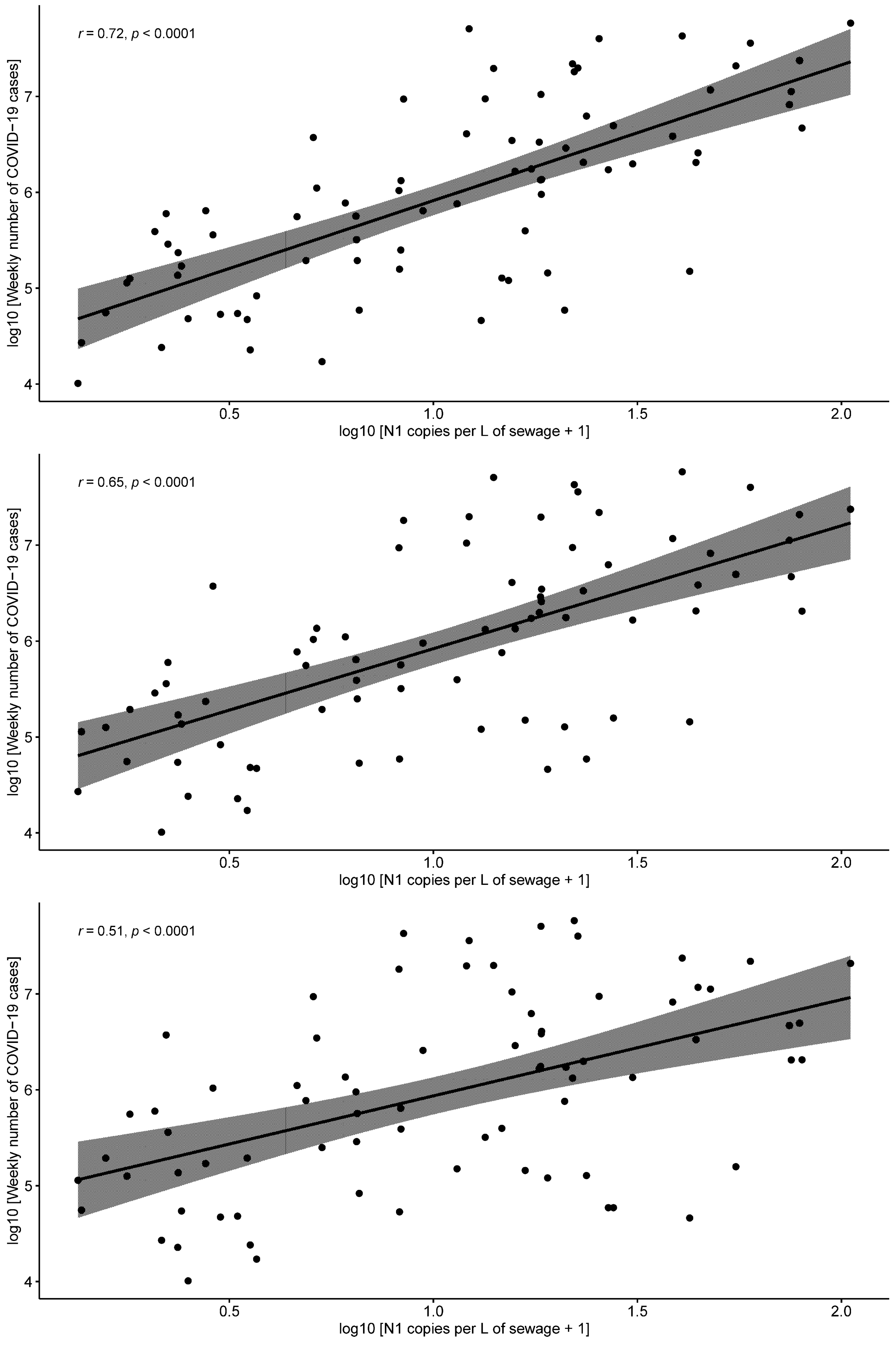

Figure S7. The correlation between sewage viral load and logged case data from July 2020 – December 2020 by weekly lag in the study area. From top to bottom: 0 week, 1 week, and 2-week lag.

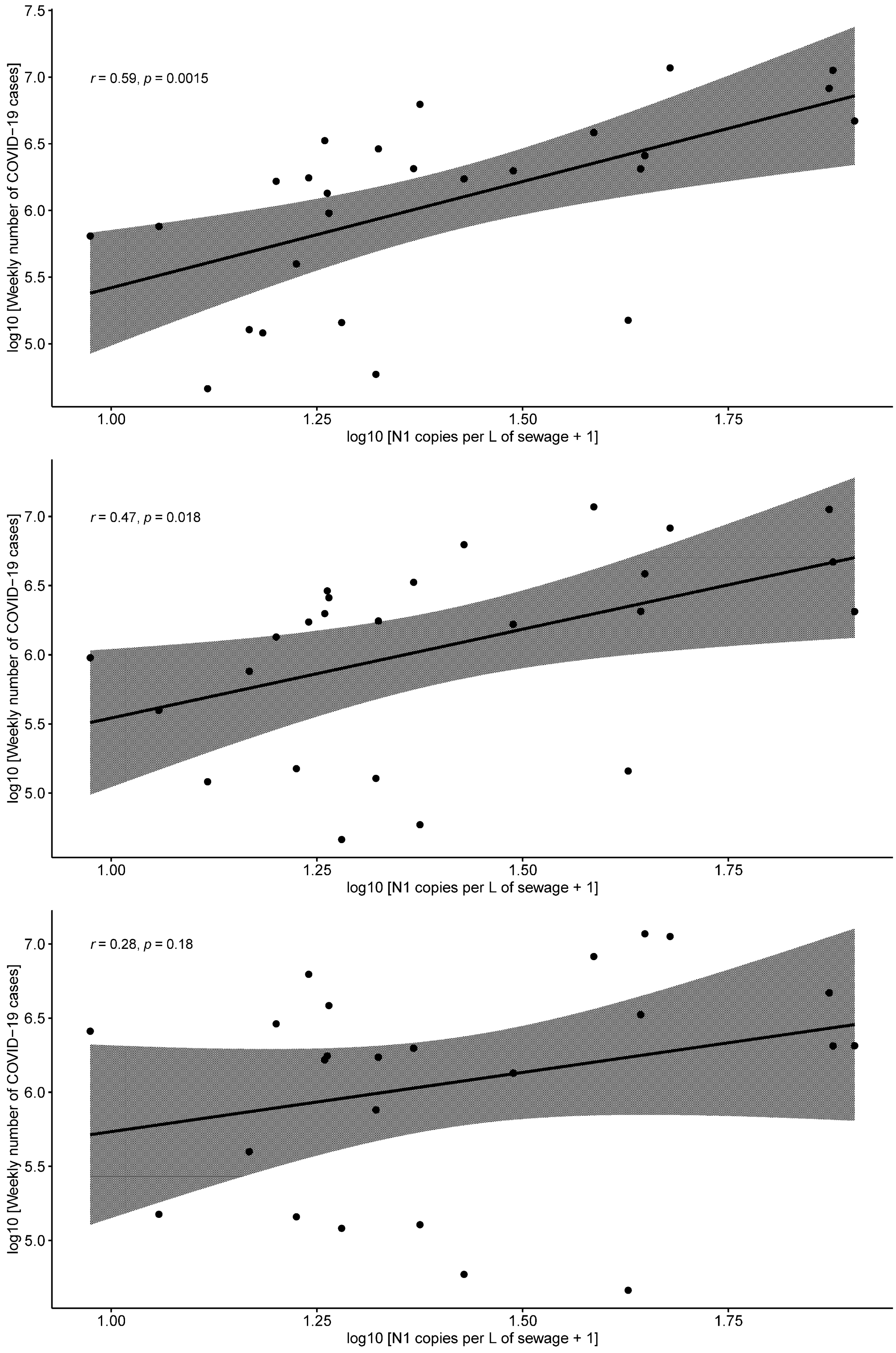

Figures S8. The correlation between sewage viral load and logged case data from January 2021 – June 2021 by weekly lag in the study area. From top to bottom: 0 week, 1 week, and 2-week lag.

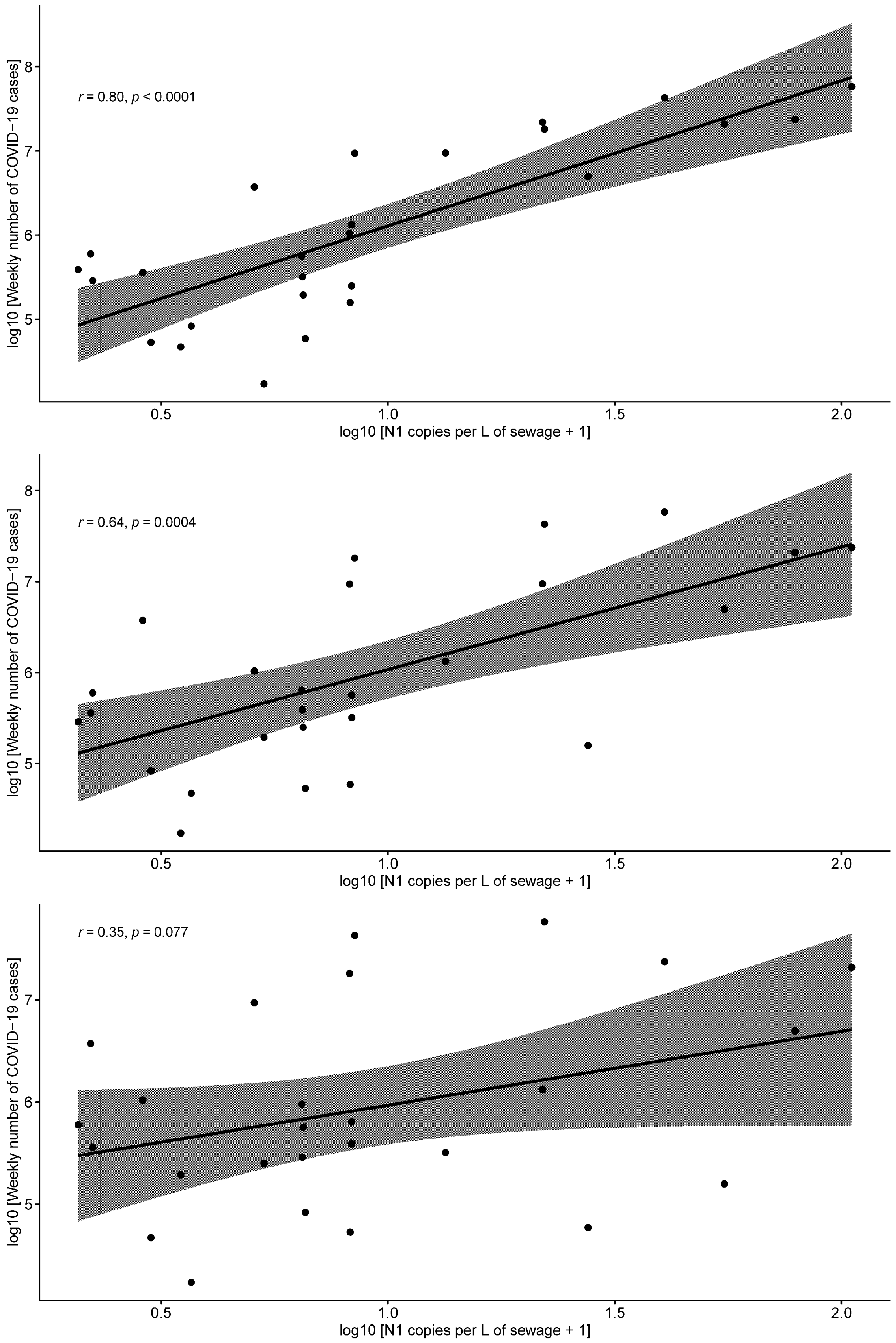

Figure S9. The correlation between sewage viral load and logged case data from July 2021 – December 2021 by weekly lag in the study area. From top to bottom: 0 week, 1 week, and 2-week lag.

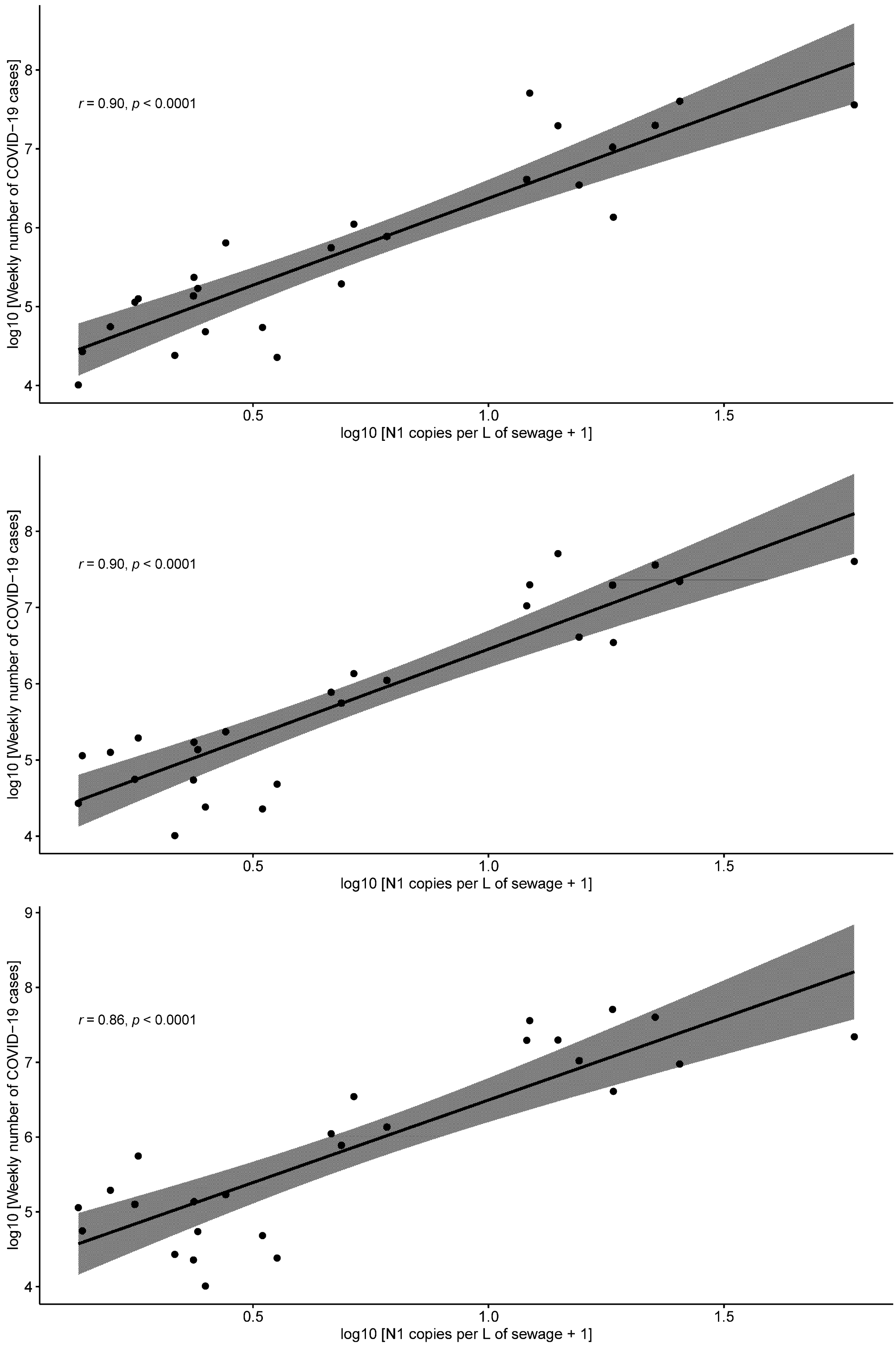

Table S3. Comparing the correlation between case data and SARS−CoV−2 viral load in environmental surveillance in the study area.

Day in lags	Increase in $\log_{10}$ cases per one $\log_{10}$ increase in viral load (95% Confidence Interval)			
	July 2020 – December 2021[a]	July 2020 – December 2020[b]	January 2021 – June 2021[c]	July 2021 – December 2021[d]
-6	0·28 (0·24, 0·31)	0·09 (0·02, 0·17)	0·27 (0·22, 0·32)	0·34 (0·28, 0·39)
-5	0·28 (0·25, 0·32)	0·10 (0·03, 0·18)	0·28 (0·23, 0·32)	0·33 (0·28, 0·39)
-4	0·29 (0·25, 0·32)	0·11 (0·04, 0·18)	0·29 (0·24, 0·33)	0·33 (0·28, 0·38)
-3	0·29 (0·26, 0·32)	0·11 (0·05, 0·18)	0·29 (0·25, 0·34)	0·33 (0·28, 0·38)
-2	0·29 (0·26, 0·33)	0·11 (0·05, 0·18)	0·30 (0·25, 0·34)	0·33 (0·28, 0·38)
-1	0·29 (0·26, 0·33)	0·12 (0·05, 0·18)	0·30 (0·26, 0·35)	0·32 (0·28, 0·37)
0	0·29 (0·26, 0·33)	0·12 (0·05, 0·18)	0·31 (0·27, 0·36)	0·32 (0·27, 0·37)
1	0·29 (0·26, 0·33)	0·12 (0·05, 0·19)	0·32 (0·27, 0·36)	0·32 (0·27, 0·36)
2	0·29 (0·26, 0·33)	0·11 (0·05, 0·18)	0·32 (0·28, 0·36)	0·32 (0·27, 0·36)
3	0·30 (0·26, 0·33)	0·11 (0·05, 0·18)	0·33 (0·29, 0·37)	0·31 (0·26, 0·36)
4	0·30 (0·26, 0·33)	0·12 (0·05, 0·18)	0·33 (0·29, 0·37)	0·31 (0·26, 0·36)
5	0·30 (0·27, 0·33)	0·12 (0·05, 0·18)	0·34 (0·30, 0·38)	0·30 (0·26, 0·35)
6	0·30 (0·27, 0·33)	0·12 (0·05, 0·19)	0·34 (0·31, 0·38)	0·30 (0·26, 0·35)
7	0·30 (0·27, 0·33)	0·13 (0·07, 0·19)	0·34 (0·31, 0·38)	0·30 (0·26, 0·35)
8	0·30 (0·27, 0·33)	0·13 (0·07, 0·19)	0·34 (0·30, 0·37)	0·30 (0·25, 0·34)
9	0·29 (0·26, 0·33)	0·13 (0·07, 0·19)	0·33 (0·30, 0·37)	0·30 (0·25, 0·34)
10	0·29 (0·26, 0·32)	0·13 (0·06, 0·19)	0·33 (0·30, 0·37)	0·29 (0·25, 0·33)
11	0·29 (0·26, 0·32)	0·13 (0·07, 0·20)	0·33 (0·29, 0·36)	0·29 (0·24, 0·33)
12	0·28 (0·25, 0·31)	0·14 (0·07, 0·20)	0·32 (0·28, 0·35)	0·28 (0·24, 0·32)
13	0·28 (0·25, 0·31)	0·14 (0·08, 0·20)	0·31 (0·28, 0·35)	0·28 (0·24, 0·32)
14	0·27 (0·24, 0·30)	0·14 (0·08, 0·20)	0·31 (0·27, 0·34)	0·27 (0·23, 0·31)
15	0·27 (0·24, 0·29)	0·14 (0·08, 0·20)	0·29 (0·26, 0·33)	0·27 (0·23, 0·31)
16	0·26 (0·23, 0·29)	0·14 (0·08, 0·20)	0·29 (0·25, 0·32)	0·26 (0·22, 0·30)
17	0·25 (0·23, 0·28)	0·14 (0·08, 0·19)	0·28 (0·25, 0·31)	0·25 (0·21, 0·29)
18	0·24 (0·22, 0·27)	0·14 (0·08, 0·19)	0·27 (0·24, 0·30)	0·24 (0·20, 0·28)
19	0·23 (0·21, 0·26)	0·13 (0·08, 0·19)	0·25 (0·22, 0·29)	0·23 (0·19, 0·27)
20	0·22 (0·19, 0·24)	0·13 (0·07, 0·18)	0·24 (0·20, 0·27)	0·22 (0·18, 0·26)
21	0·21 (0·18, 0·23)	0·13 (0·07, 0·18)	0·22 (0·18, 0·25)	0·21 (0·17, 0·25)

These data also reported in Figure 4 and Figure S12. [a] rho = 0.15 (5-day lag). [b] rho = 0.12 (15-day lag). [c] rho = 0.22 (6-day lag). [d] rho = 0.17 (0-day lag).

Figure S10. Cross-correlations comparing the correlation between COVID-19 clinical case data and SARS-CoV-2 viral load in environmental surveillance data from July 2020-December 2021 from the study area. Black horizontal dashed line indicates the level of no significance. Day lag indicates the number of days before (negative) or after (positive) the environmental sample was collected where day 0 represents the day of sample collection. The clinical case data is from the 8 wards where the 37 catchment sites are located.

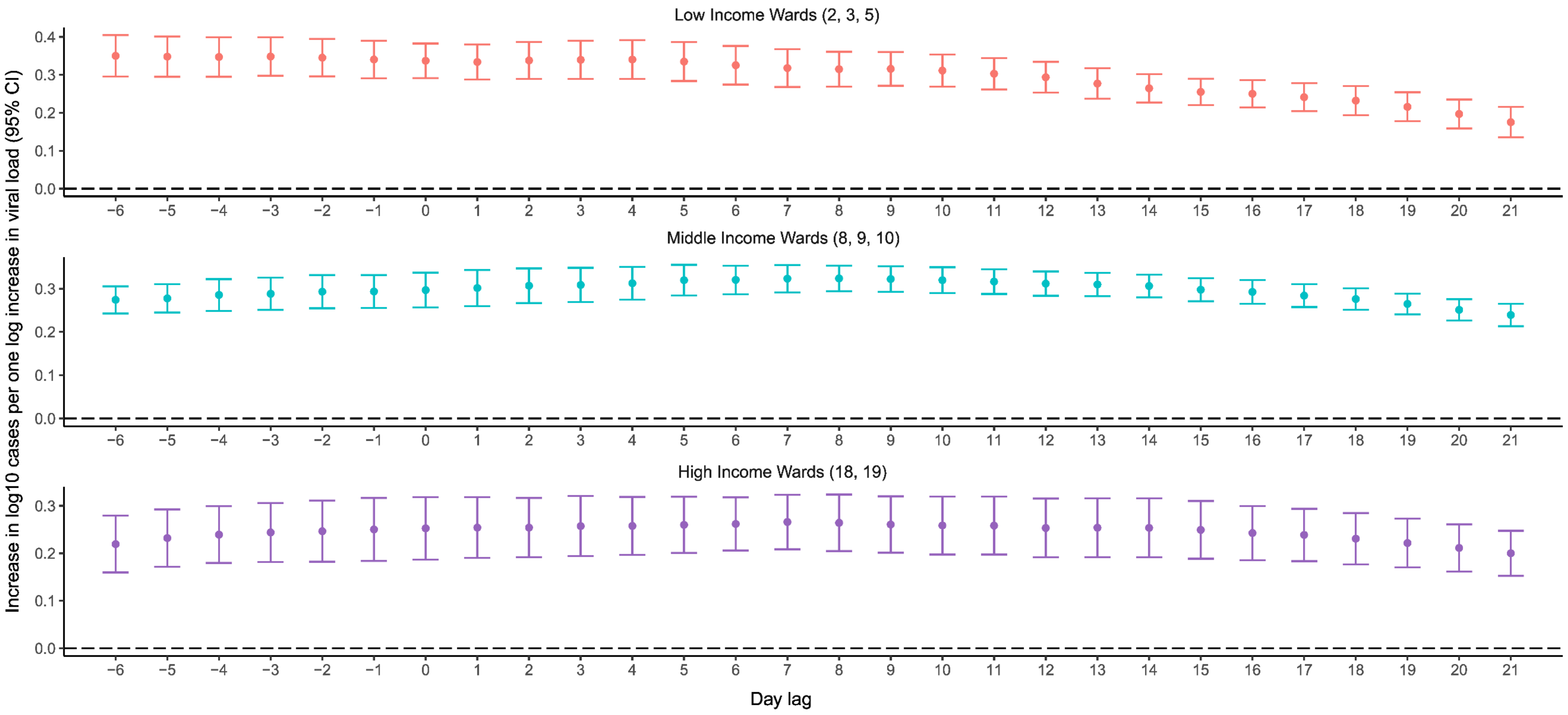

Supplement: Supplementary appendix [file mmc1.pdf]
